# Intestinal gluconeogenesis shapes gut microbiota, fecal and urine metabolome in mice with gastric bypass surgery

**DOI:** 10.1038/s41598-022-04902-y

**Published:** 2022-01-26

**Authors:** Justine Vily-Petit, Aude Barataud, Carine Zitoun, Amandine Gautier-Stein, Matteo Serino, Gilles Mithieux

**Affiliations:** 1 INSERM UMR-S1213, Université Claude Bernard Lyon 1, Université de Lyon, Lyon, France; 2 INSERM, INRAE, ENVT, UPS, IRSD, Université de Toulouse, Toulouse, France

**Keywords:** Physiology, Metabolism, Metabolic diseases, Obesity

## Abstract

Intestinal gluconeogenesis (IGN), gastric bypass (GBP) and gut microbiota positively regulate glucose homeostasis and diet-induced dysmetabolism. GBP modulates gut microbiota, whether IGN could shape it has not been investigated. We studied gut microbiota and microbiome in wild type and IGN-deficient mice, undergoing GBP or not, and fed on either a normal chow (NC) or a high-fat/high-sucrose (HFHS) diet. We also studied fecal and urine metabolome in NC-fed mice. IGN and GBP had a different effect on the gut microbiota of mice fed with NC and HFHS diet. IGN inactivation increased abundance of *Deltaproteobacteria* on NC and of *Proteobacteria* such as *Helicobacter* on HFHS diet. GBP increased abundance of *Firmicutes* and *Proteobacteria* on NC-fed WT mice and of *Firmicutes*, *Bacteroidetes* and *Proteobacteria* on HFHS-fed WT mice. The combined effect of IGN inactivation and GBP increased abundance of *Actinobacteria* on NC and the abundance of *Enterococcaceae* and *Enterobacteriaceae* on HFHS diet. A reduction was observed in the amounf of short-chain fatty acids in fecal (by GBP) and in both fecal and urine (by IGN inactivation) metabolome. IGN and GBP, separately or combined, shape gut microbiota and microbiome on NC- and HFHS-fed mice, and modify fecal and urine metabolome.

## Introduction

The worldwide rise in the prevalence of obesity is associated with increased incidence of various metabolic disorders. In this context, bariatric surgeries have emerged as the most effective therapies to treat obesity and one of its comorbidities such as type 2 diabetes mellitus (T2DM)^1,2^. The Roux-en-Y gastric bypass (GBP) procedure is one of the most performed and efficient bariatric surgeries. After GBP, patients exhibit reduced food intake and a considerable and long-term weight loss of up to 30%^2,3^. Improvement or even remission of T2DM and its associated complications as hepatic steatosis and fibrosis is observed in most patients^3,4^. However, the improvements in glycaemic control cannot be exclusively correlated to weight loss. Indeed, many type 2 diabetic patients stop their medication within days after surgery, before any significant body weight loss occurred^1,2^. Thus, a better understanding of mechanisms underlying metabolic improvements initiated by GBP has been the matter of huge efforts by the scientific community over the last decades. Given the role of gut microbiota in metabolic diseases, it has been often suggested that a modification in gut microbiota composition could have a role in the metabolic benefits associated with GBP^5,6^.

Among other mechanisms proposed to account for metabolic benefits of GBP, one relates to the activation of intestinal gluconeogenesis (IGN), documented by several groups^7–10^. An increased IGN was proven to induce beneficial effects on glucose homeostasis and energy metabolism at the hypothalamic level^11–13^. Glucose deriving from IGN is released into the portal vein and sensed by a portal glucose sensor, which initiates a gut-brain-liver circuit inducing satiety, increased hepatic insulin sensitivity and decreased hepatic glucose production^13^. In fact, IGN is increased and hepatic glucose production decreased in two models of GBP, i.e. duodenal-jejunal GBP in rats^7,8,10^ and enterogastroanastomosis in mice^9^. IGN has been recently documented activated after GBP in humans^14,15^. Moreover, the metabolic outcomes of GBP in obese patients are positively associated with IGN at the time of surgery^16^. It is noteworthy that, independently of GBP, IGN is markedly activated by short chain fatty acids (SCFAs), i.e. propionate and butyrate that are gut microbial products derived from fermentation of dietary fibers^11^. Hence, the activation of IGN by SCFAs allowed us to explain the anti-obesity and anti-diabetic effects of dietary fibers, the latters being comparable to the metabolic benefits of GBP surgery^11^. SCFAs are also a crucial factor of bacterial cross-feeding, which may influence the growth or decay of specific bacterial species^17^. It is noteworthy that the changes in cecal SCFAs content and composition induced by fiber-enriched diet are modulated acccording to the presence or absence of IGN^11^. Thus, it was a first question of this study to know whether IGN inactivation per se might shape gut microbiota then influencing SCFAs production. We addressed this question using IGN-deficient mice (knocked-out for the catalytic subunit of glucose-6-phosphatase specifically in the intestine (iG6PC-KO)) compared to wild-type (WT) mice.

The second question was to know whether a shift in gut microbiota to SCFAs-producing bacteria could account for metabolic benefits associated with GBP. To address this question, both WT and iG6PC-KO mice underwent GBP. Laparotomized (Lap) mice served as control group for GBP. To analyse how a shift in gut microbiota might affect host metabolites, we performed a fecal and urine metabolome analysis on normal chow (NC)-fed mice. Finally, since the type of food profoundly alters gut microbiota composition^18^, we studied both WT and iG6PC-KO mice that underwent GBP and were fed either a NC or a high-fat high-sucrose (HFHS) diet.

## Results

### Effects of intestinal gluconeogenesis inactivation on gut microbiota and microbiome in normal chow and high-fat high-sucrose fed mice

Please note first that throughout the manuscript the term *microbiota* refers to ecological structure (relative abundance in %) of gut microbes, whereas the term *microbiome* refers to microbial inferred functions.

**Figure 1:**
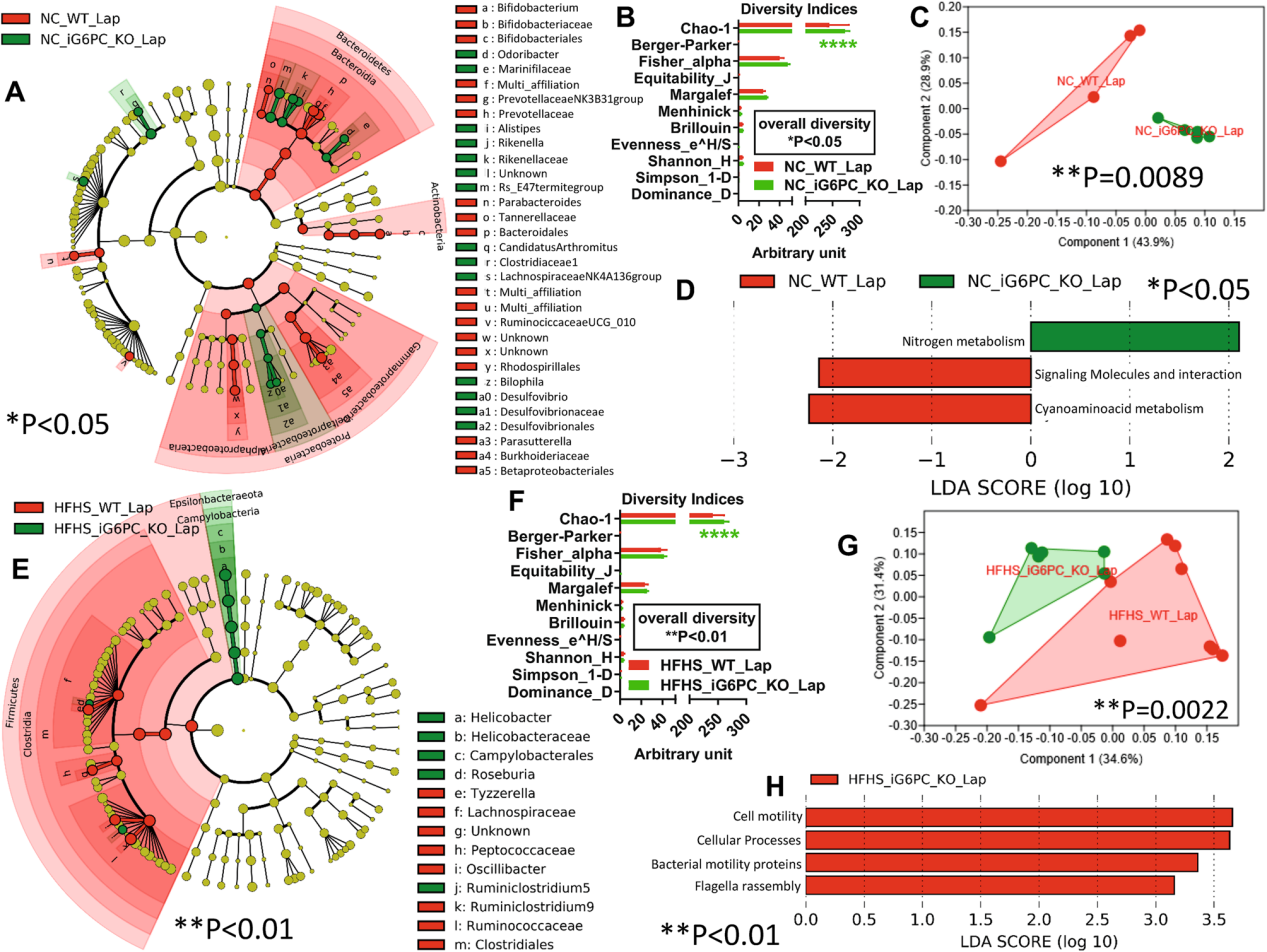
Inactivation of intestinal gluconeogenesis changes gut (caecum) microbiota and microbiome of NC- and HFHS-fed mice. (**A**,**E**) Cladogram showing bacterial taxa significantly higher in the group of mice of the same colour, in the caecal microbiota (the cladogram shows taxonomic levels represented by rings with phyla at the innermost and genera at the outermost ring and each circle is a bacterial member within that level). (**B**,**F**) Indices of gut microbiota diversity, ****P < 0.0001, 2-way ANOVA followed by a 2-stage linear step-up procedure of Benjamini, Krieger and Yekutieli to correct for multiple comparisons by controlling the False Discovery Rate (< 0.05). (**C**,**G**) Principal Component Analysis (PCA) of gut microbiota, **P < 0.01, 1-way PERMANOVA with Bonferroni correction. (**D**,**H**) LDA score for predictive microbial pathway identified via PICRUSt^40^, *P < 0.05 for D, **P < 0.01, for H with alpha value for the factorial Kruskal–Wallis test among classes and alpha value for the pairwise Wilcoxon test between subclasses set both at 0.01 and threshold on the logarithmic LDA score for discriminative features set at 3. (“Lap” stands for laporotomized). *Please note that in Fig. 1H the group HFHS_WT_Lap is not shown because this group has no microbial functional pathways enriched compared to the group HFHS_iG6PC_KO_Lap, which, hence, is shown in red. “*n” for: NC_WT_Lap = 4, NC_iG6PC_KO_Lap = 5, HFHS_WT_Lap = 9 and HFHS_iG6PC_KO_Lap = 6.

We assessed the effect of IGN inactivation on both gut microbiota and microbiome in NC-fed mice (these mice underwent a laparotomy (identified as “Lap” group) to serve as control for mice that underwent GBP). On a NC, iG6PC-KO mice had a statistically significant higher relative abundance of bacteria from phylum *Proteobacteria*, such as *Desulfovibrio*, and from phylum *Bacteroidetes*, such as *Odoribacter*, *Alistipes*, *Rikenella* as well as bacteria from phylum *Firmicutes* such as *Candidatus Arthromitus*, compared to WT mice (Fig. 1A). The overall diversity of gut microbiota of NC-fed iG6PC-KO was significantly different from that of WT mice, mostly based on Chao-1 index (Fig. 1B) as confirmed by comparison of overall microbial profiles (Fig. 1C). In addition, the microbiome of NC-fed iG6PC-KO exhibited an imputed functional statistically significant increase of nitrogen metabolism (Fig. 1D).

Then, we studied the impact of IGN inactivation on gut microbiota and microbiome of both WT and iG6PC-KO mice fed a high-fat/high-sucrose (HFHS) diet. The major change was the statistically significant higher abundance of genus *Helicobacter* (*Proteobacteria* phylum) in the gut microbiota of HFHS-fed iG6PC-KO mice compared to WT mice (Fig. 1E). The overall diversity of gut microbiota of HFHS-fed iG6PC-KO was significantly different from that of WT mice, mostly based on Chao-1 index (Fig. 1F) as confirmed by comparison of overall microbial profiles (Fig. 1G). In addition, the microbiome of HFHS-fed iG6PC-KO showed two statistically significant higher bacterial motility-related inferred functions (Fig. 1H). Altogether, these data indicate that IGN inactivation affects gut microbiota composition and functions regardless of diet.

### Intestinal gluconeogenesis inactivation reduces acetate in fecal and urine metabolome

**Figure 2:**
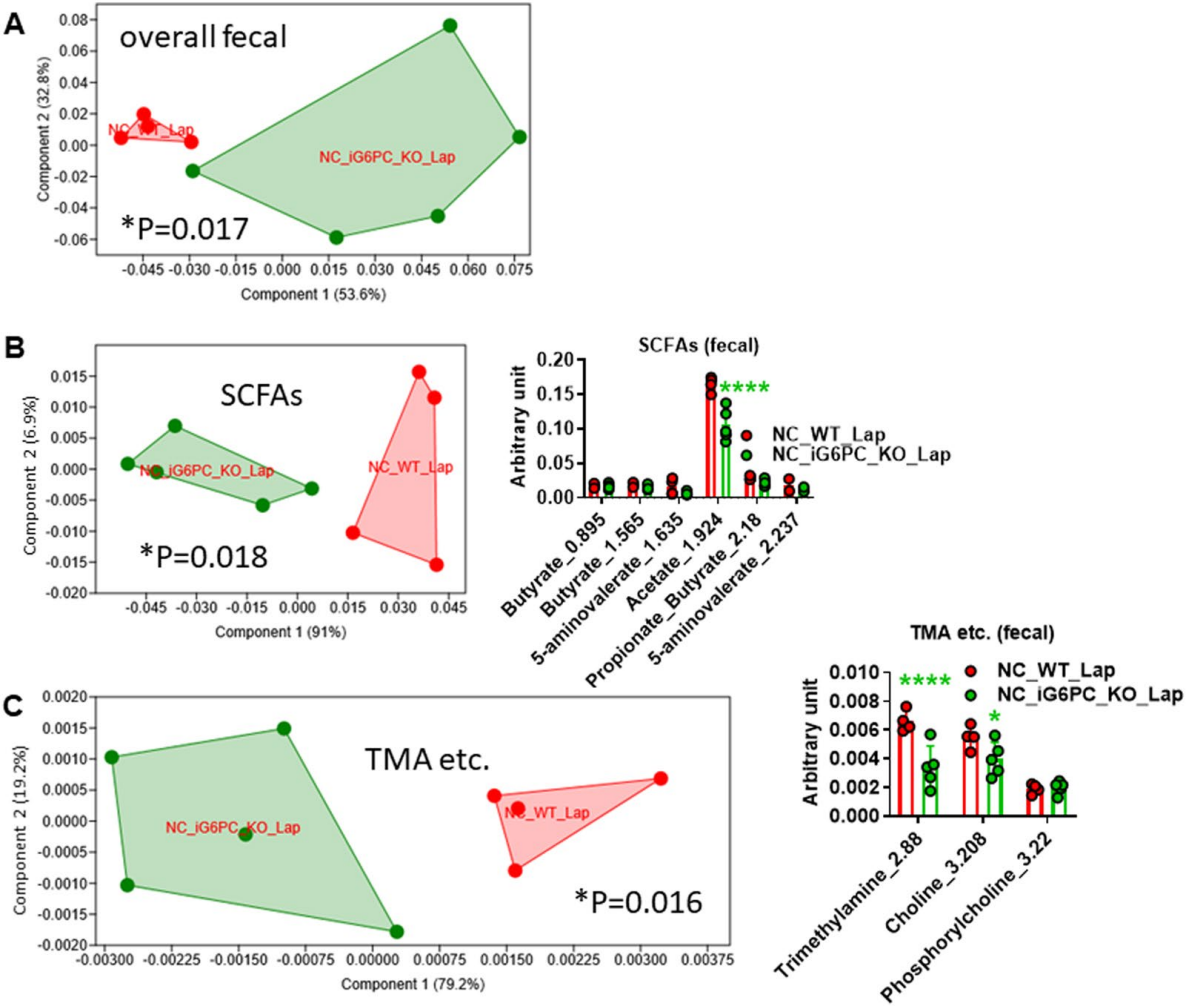
Metabolomic analysis in feces from NC-fed WT and i G6PC-KO mice. PCA of: (**A**) overall fecal metabolome, (**B**) short chain fatty acids (SCFAs), (**C**) trimethylamine (TMA) and other related metabolites. For PCA, 1-way PERMANOVA with Bonferroni correction; for hystograms (**B**, **C**), *P < 0.05, ****P < 0.0001, 2-way ANOVA followed by a 2-stage linear step-up procedure of Benjamini, Krieger and Yekutieli to correct for multiple comparisons by controlling the False Discovery Rate (< 0.05). “n” for: NC_WT_Lap = 4, NC_iG6PC_KO_Lap = 5.

**Figure 3:**
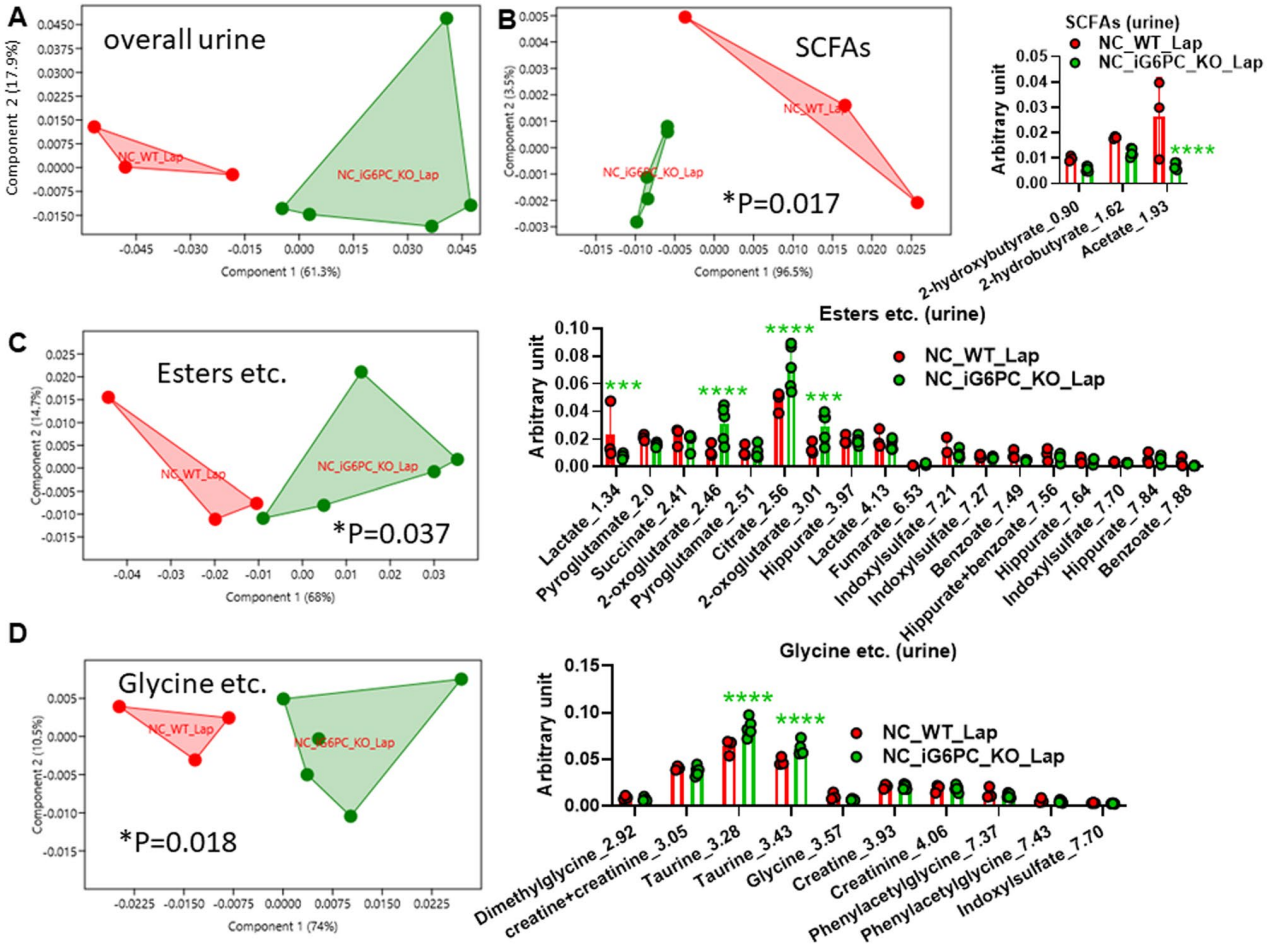
Metabolomic analysis in urines from NC-fed WT and i G6PC-KO mice. PCA of: (**A**) overall urine metabolome, (**B**) SCFAs, (**C**) esters and other metabolites, (**D**) glycine and other metabolites. For PCA, 1-way PERMANOVA with Bonferroni correction; for hystograms (**B**-**D**), ***P < 0.001, ****P < 0.0001, 2-way ANOVA followed by a 2-stage linear step-up procedure of Benjamini, Krieger and Yekutieli to correct for multiple comparisons by controlling the False Discovery Rate (< 0.05). “n” for: NC_WT_Lap = 3, NC_iG6PC_KO_Lap = 5.

Since IGN inactivation affects gut microbiota composition and functions on both NC- and HFHS-feeding, as reported above, and to avoid masking effects of HFHS diet on iG6PC-KO genotype^19^, we evaluated the impact of IGN inactivation on both fecal and urine metabolome in NC-fed mice only. The overall fecal metabolome profile of iG6PC-KO mice was statistically significantly different from that of WT mice (Fig. 2A), due to a significant reduction in the levels of acetate (Fig. 2B), and trimethylamine (TMA) and its precursor choline (Fig. 2C). Other changes were observed in the amount of fecal sugars, amino acids and of esters and other metabolites, without a significant impact on the overall profile (Supplementary Fig. 1). The overall urine metabolome profile was not significantly affected by IGN inactivation though the amounf of SCFAs, esters and other metabolites showed statistically significant different profiles from those of WT mice (Fig. 3). The amount of TMA and its precursors together with urine volume out of 48 h did not significantly vary (Supplementary Fig. 2A,B). These data were associated with an increased food intake over 48 h (Supplementary Fig. 2C) with no significant effect on body weight (Supplementary Fig. 2D). Altogether, these data indicate that IGN inactivation affects both fecal and urine metabolome.

### Effects of gastric bypass on gut microbiota, microbiome, fecal and urine metabolome in normal chow fed mice

**Figure 4:**
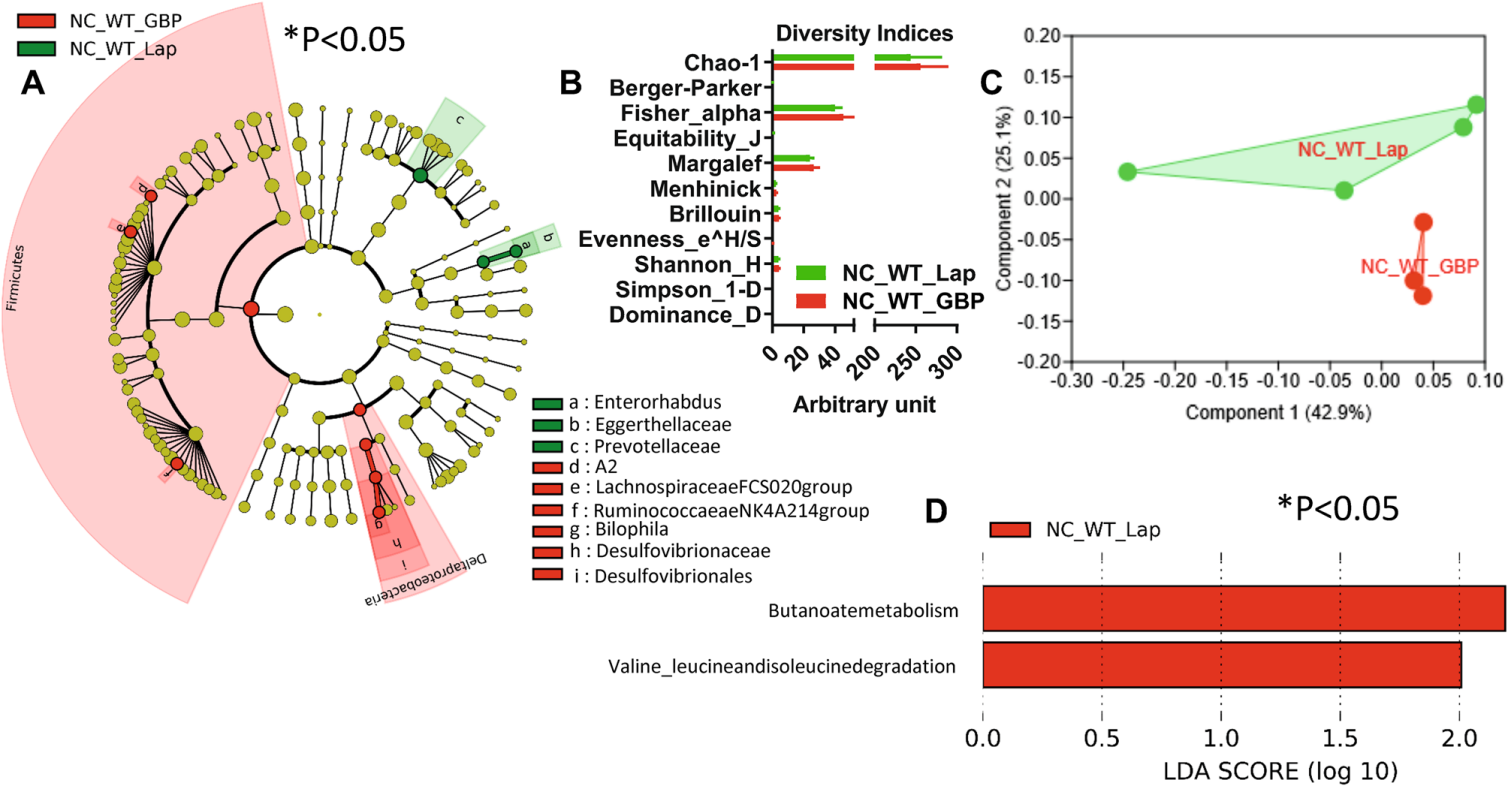
Gastric bypass changes gut (caecum) microbiota and microbiome in NC-fed WT mice. (**A**) Cladogram showing bacterial taxa significantly higher in the group of mice of the same colour, in the caecal microbiota. (**B**) Indices of gut microbiota diversity. (**C**) PCA of gut microbiota. (**D**) LDA score for predictive microbial pathway identified via PICRUSt^40^. (“Lap” stands for laporotomized; “PF” stands for pair-feeding). “n” for: NC_WT_Lap = 4; NC_WT_GBP = 3. *Please note that in Fig. 4D the group NC_WT_GBP is not shown because this group has no microbial functional pathways enriched compared to the group NC_WT_Lap, which, hence, is shown in red.* “n” for: NC_WT_GBP = 3 and for NC_WT_Lap = 4.

**Figure 5:**
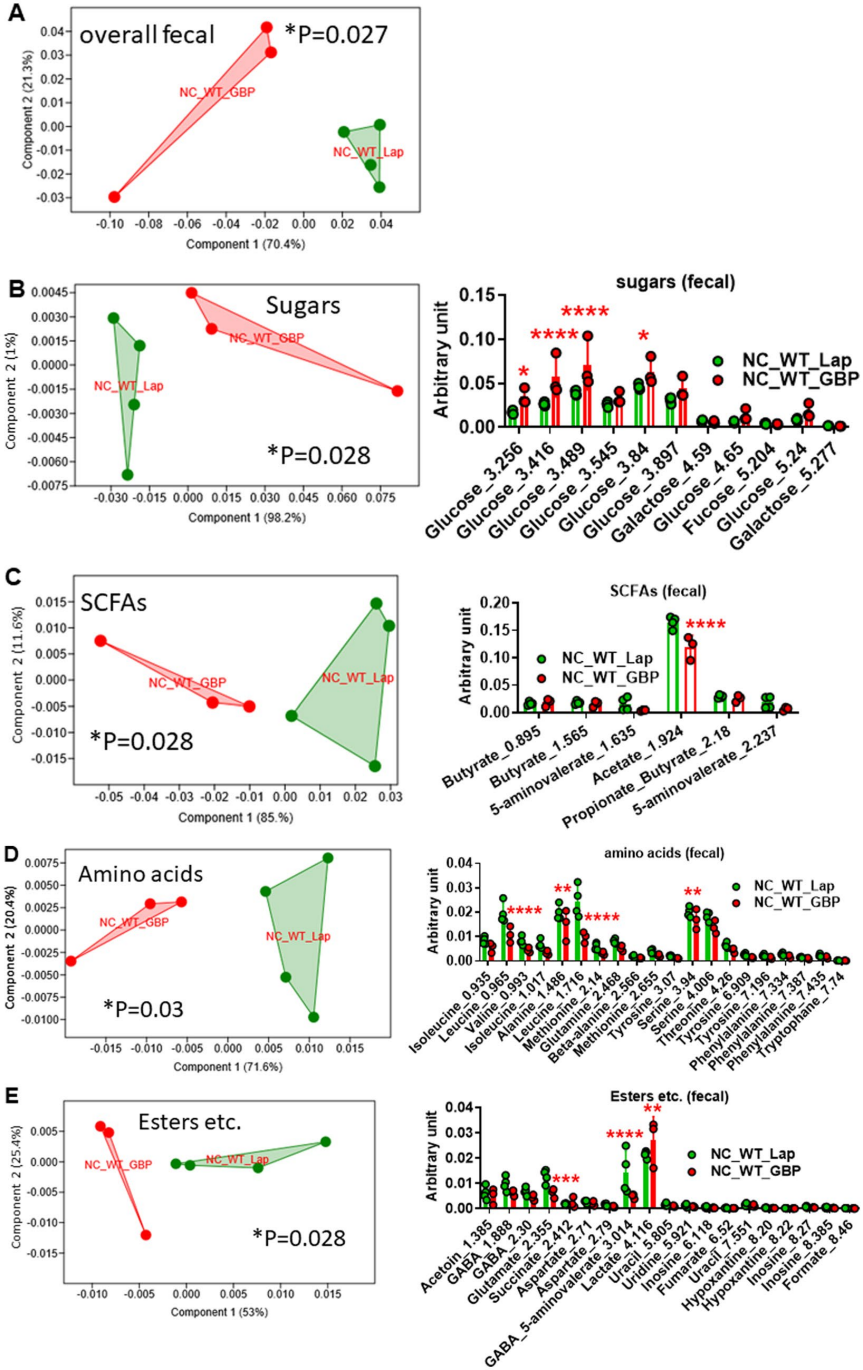
Metabolomic analysis in feces from NC-fed WT mice following gastric bypass. PCA of: (**A**) overall fecal metabolome, (**B**) sugars, (**C**) SCFAs, (**D**) amino acids, (**E**) esters and other metabolites. For PCA, 1-way PERMANOVA with Bonferroni correction; for hystograms (**B**-**E**), *P < 0.05, **P < 0.01, ***P < 0.001, ****P < 0.0001, 2-way ANOVA followed by a 2-stage linear step-up procedure of Benjamini, Krieger and Yekutieli to correct for multiple comparisons by controlling the False Discovery Rate (< 0.05). “n” for: NC_WT_Lap = 4, NC_WT_GBP = 3.

GBP is one of the most promising and effective treatments for severe obesity, also inducing a huge amelioration of T2DM. To understand the impact of GBP on gut microbiota, we analysed both gut microbiota and microbiome in NC- and HFHS-fed WT mice after GBP surgery. GBP was previously reported not to change food intake on NC^20,21^, thus, there was no rationale to study a pair-fed (PF) group. On NC, GBP induced a statistically significant shift of gut microbiota towards *Firmicutes* and *Deltaproteobacteria* (Fig. 4A) compared to control mice that underwent laparotomy. This shift was associated with a microbiome significantly different between groups (Fig. 4D). The overall diversity (Fig. 4B) and gut microbiota profile (Fig. 4C) were not statistically different. GBP induced a significant change in overall fecal metabolome (Fig. 5A), due to increased fecal glucose (Fig. 5B), reduced fecal acetate content (Fig. 5C), a general significant reduction in fecal amino acid content (Fig. 5D) and a significant change in the amount of several esters (Fig. 5E) with no significant change in the amount of TMA and its precursors (Supplementary Fig. 3A). By contrast, GBP slightly affected urine metabolome (Supplementary Fig. 3B-FB-F) with no change in urine volume over 24 and 48 h (Supplementary Fig. 3G).

### Gastric bypass modifies gut microbiota and microbiome in high-fat high-sucrose fed mice

**Figure 6:**
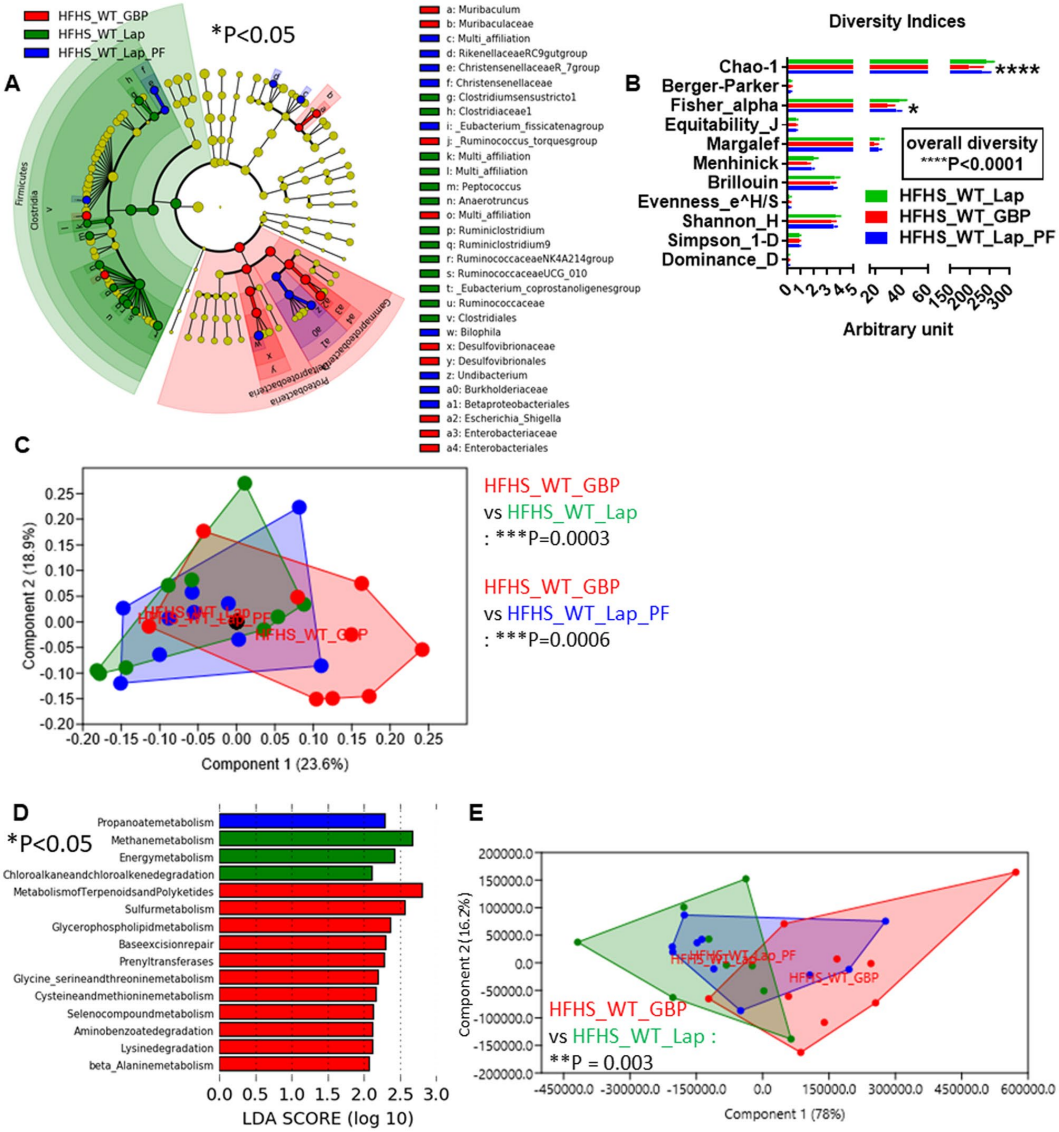
Gastric bypass changes gut (caecum) microbiota and microbiome of HFHS-fed mice. (**A**) Cladogram showing bacterial taxa significantly higher in the group of mice of the same colour, in the caecal microbiota. (**B**) Indices of gut microbiota diversity, *P < 0.01 and ****P < 0.0001, 2-way ANOVA followed by a 2-stage linear step-up procedure of Benjamini, Krieger and Yekutieli to correct for multiple comparisons by controlling the False Discovery Rate (< 0.05). C) PCA of the gut microbiota, ***P < 0.001, 1-way PERMANOVA with Bonferroni correction. (**D**) LDA score for predictive microbial pathway identified via PICRUSt^40^. (“Lap” stands for laporotomized; “PF” stands for pair-feeding). (**E**) PCA of the gut microbiome, **P < 0.01, 1-way PERMANOVA with Bonferroni correction. Data used to generate this PCA are those reported in Fig. 6D to generate LDA score. “n” for: HFHS_WT_Lap = 9; HFHS_WT_Lap_PF = 10; HFHS_WT_GBP = 9.

GBP was reported to decrease food intake during HFHS feeding^22,23^. Thus, to avoid the impact of decreased food intake on gut microbiota, we set up a group of pair-fed (PF) WT mice (HFHS_WT_Lap_PF mice) that underwent laparotomy and received the same amount of food as GBP mice. During HFHS feeding, we observed that GBP in WT mice induced a statistically significant shift towards *Proteobacteria*, compared to both WT-Lap and WT-Lap-PF mice (Fig. 6A). The overall diversity was significantly highly different, based on Chao-1 and Fisher-alpha indices (Fig. 6B) as well as the general gut microbial profile (Fig. 6C). GBP also induced a statistically significant increase in many inferred microbial functions related to chemicals metabolism (Fig. 6D) with an overall microbiome profile significantly different from that of control mice not PF (Fig. 6E).

### Combined effect of GBP and IGN inactivation on gut microbiota, microbiome, fecal and urine metabolome in normal chow fed mice

**Figure 7:**
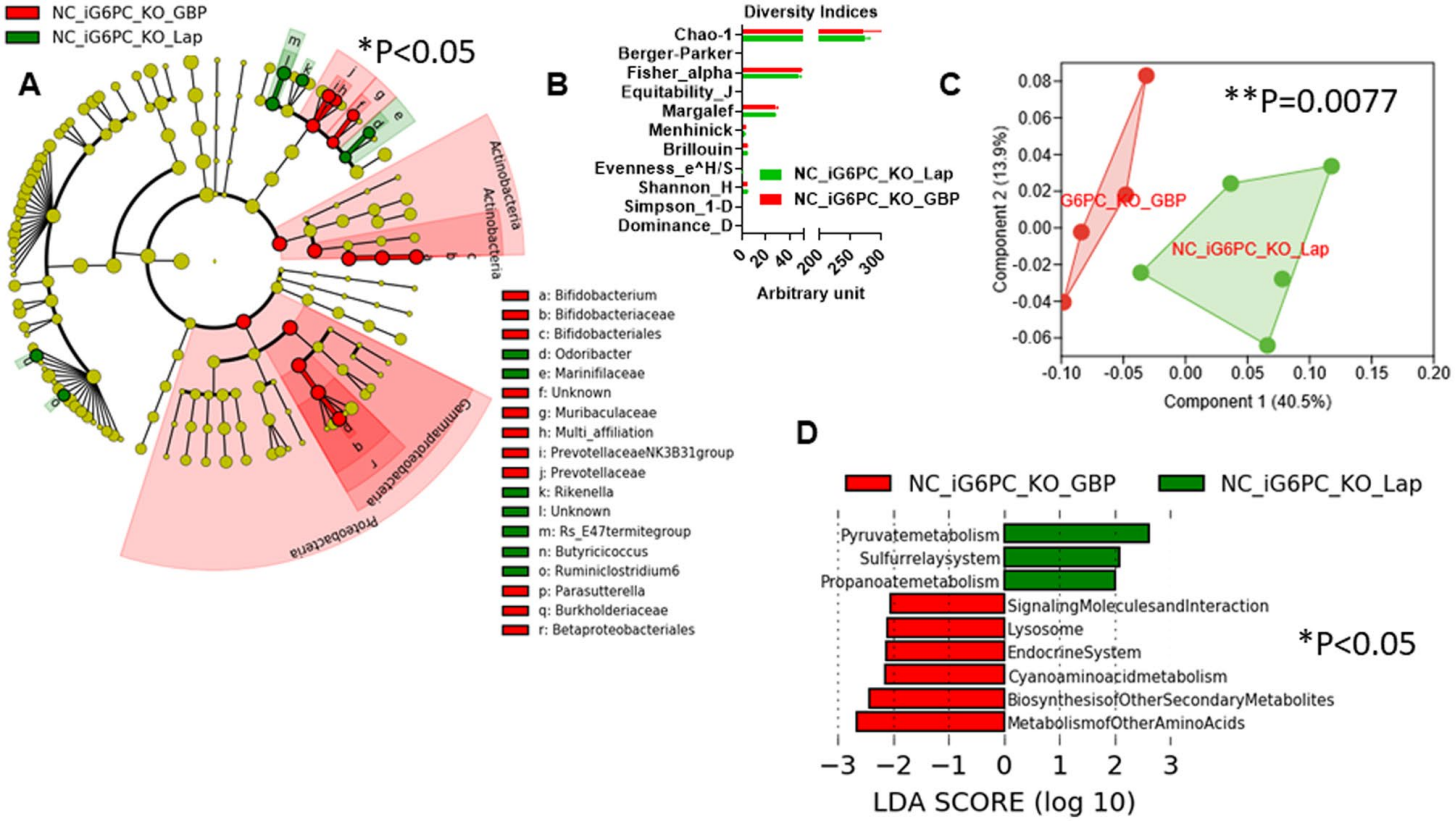
Combined impact of intestinal gluconeogenesis inactivation and gastric bypass on gut (caecum) microbiota and microbiome of NC-fed mice. (**A**) Cladogram showing bacterial taxa significantly higher in the group of mice of the same colour, in the caecal microbiota. (**B**) Indices of gut microbiota diversity. (**C**) PCA of the gut microbiota, **P < 0.01, 1-way PERMANOVA with Bonferroni correction. (**D**) LDA score for predictive microbial pathway identified via PICRUSt^40^, *P < 0.05. (“Lap” stands for laporotomized; “PF” stands for pair-feeding). “n” for: NC_iG6PC_KO_GBP = 4, NC_iG6PC_KO_Lap = 5.

Next, we evaluated the combined effect of GBP and IGN inactivation on gut microbiota, microbiome, fecal and urine metabolome during NC. iG6PC-KO mice that underwent GBP showed a statistically significant higher abundance of *Proteobacteria* and *Actinobacteria*, compared to Lap iG6PC-KO mice (Fig. 7A). None of the diversity indices was significantly changed (Fig. 7B), though the overall gut microbiota profiles were significantly dissimilar (Fig. 7C) and the microbiome of iG6PC-KO mice that underwent GBP showed biggest significant changes in pathways related to amino acids and cyano amino acids metabolism and biosynthesis of secondary metabolites (Fig. 7D). Note that iG6PC-KO mice did affect neither food intake nor body weight (Supplementary Fig. 4). These data indicate that GBP may counteract IGN inactivation-induced change in gut microbiota.

**Figure 8:**
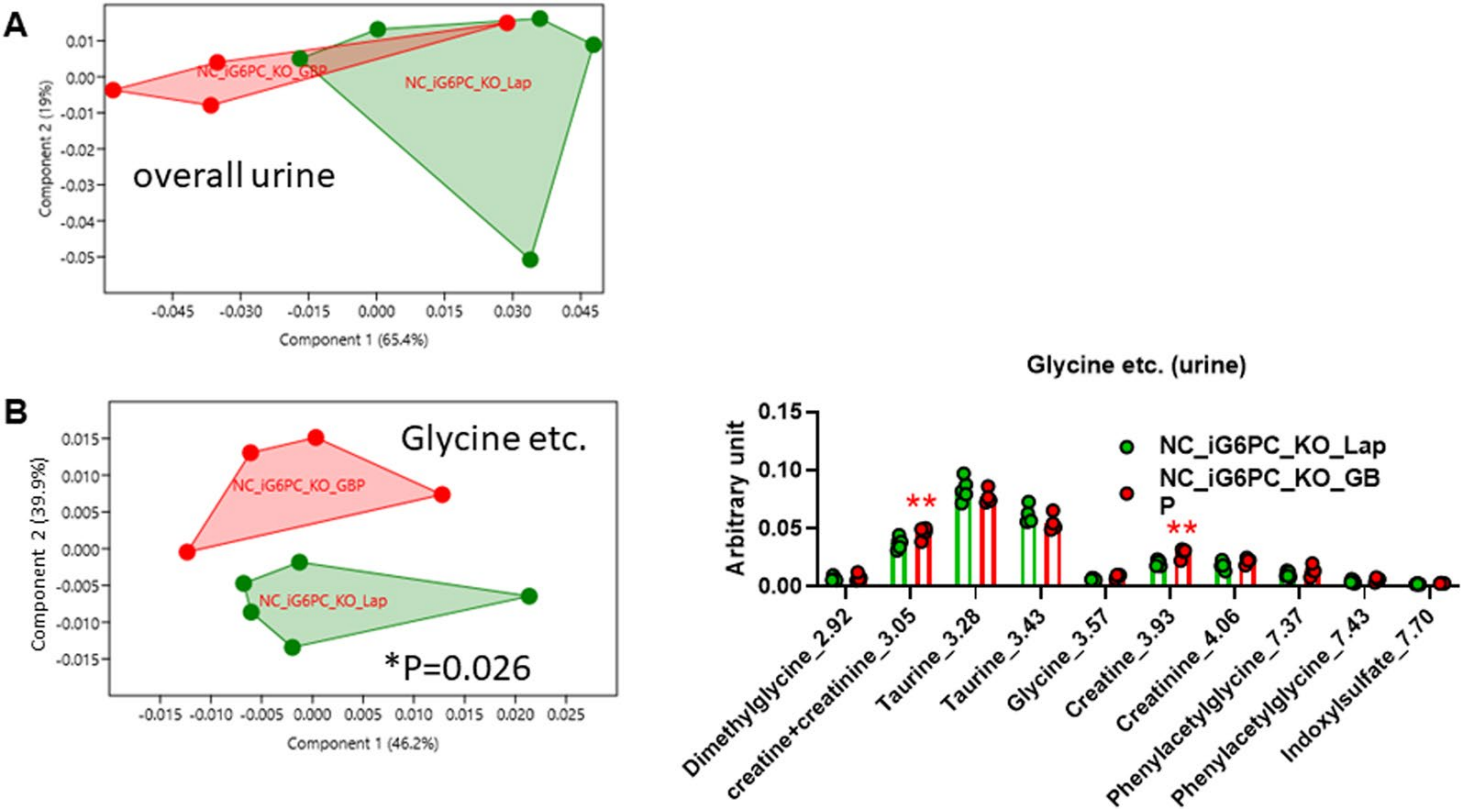
Metabolomic analysis in urine from NC-fed i G6PC-KO mice following gastric bypass. PCA of: (**A**) overall urine metabolome, (**B**) glycine and other metabolites. For PCA, 1-way PERMANOVA with Bonferroni correction; for histogram (**B**), **P < 0.01, 2-way ANOVA followed by a 2-stage linear step-up procedure of Benjamini, Krieger and Yekutieli to correct for multiple comparisons by controlling the False Discovery Rate (< 0.05). “n” for: NC_iG6PC_KO_Lap = 5, NC_iG6PC_KO_GBP = 4.

It is noteworthy that in iG6PC-KO mice GBP did not significantly change fecal metabolome (Supplementary Fig. 5) and slightly significantly changed urine metabolome (Fig. 8A,B and Supplementary Fig. 6).

We did not identify any cluster of parameters from gut microbiota, microbiome and fecal metabolites (Supplementary Fig. 7A). By contrast, the genus *Lactobacillus* was significantly and positively correlated with microbial pathway related to transcription proteins and urine succinate (Supplementary Fig. 7B-D).

### Combined effect of GBP and IGN inactivation on gut microbiota and microbiome in HFHS-fed mice

**Figure 9:**
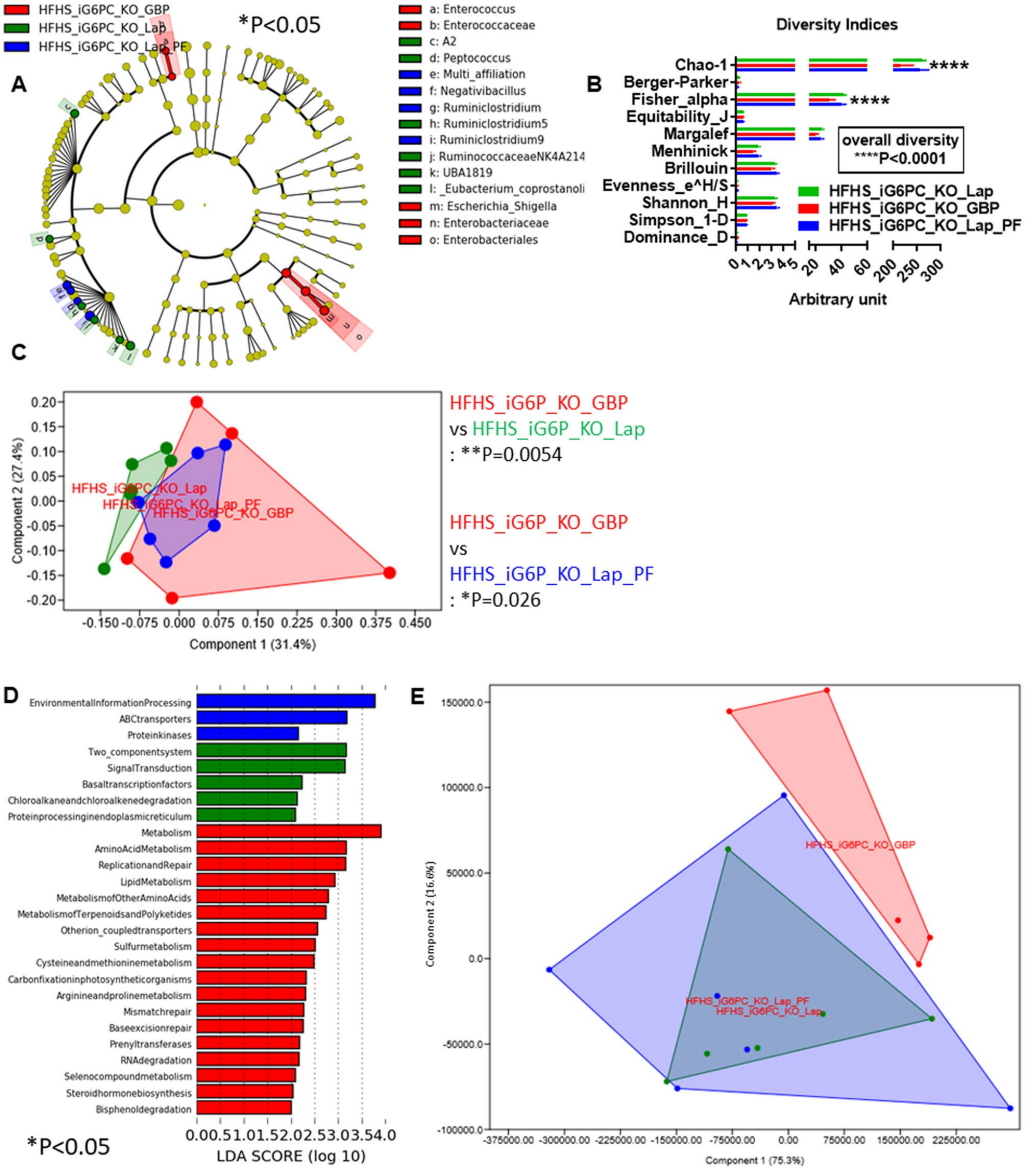
Combined impact of intestinal gluconeogenesis inactivation and gastric bypass on gut (caecum) microbiota and microbiome of HFHS-fed mice. (**A**) Cladogram showing bacterial taxa significantly higher in the group of mice of the same colour, in the caecal microbiota. (**B**) Indices of gut microbiota diversity, ****P < 0.0001, 2-way ANOVA followed by a 2-stage linear step-up procedure of Benjamini, Krieger and Yekutieli to correct for multiple comparisons by controlling the False Discovery Rate (< 0.05). (**C**) PCA of the gut microbiota, *P < 0.05, **P < 0.01, 1-way PERMANOVA with Bonferroni correction. (**D**) LDA score for predictive microbial pathway identified via PICRUSt^40^. (**E**) PCA of the gut microbiome. Data used to generate this PCA are those reported in Fig. 9D to generate LDA score. (“Lap” stands for laporotomized; “PF” stands for pair-feeding). “n” for: HFHS_iG6PC_KO_GBP = 5, HFHS_iG6PC_KO_Lap = 6, HFHS_iG6PC_KO_Lap_PF = 6.

On HFHS, iG6PC-KO mice that underwent GBP showed a statistically significant higher relative abundance of bacteria from family *Enterococcaceae* and from order *Enterobacteriales*, compared to both laparotomized iG6PC-KO mice and PF laparotomized iG6PC-KO mice (Fig. 9A). The overall diversity, mostly based on Chao-1 and Fisher-alpha indices, as well as the general gut microbiota profile of iG6PC-KO mice that underwent GBP were significantly different from the other groups of mice (Fig. 9B-C). Microbial inferred functions related to amino acid metabolism were also significantly enriched (Fig. 9D), though the general microbiome profile was not significantly divergent (Fig. 9E), compared to the other groups of mice. All these data showed that GBP in iG6PC-KO mice is able to modulate gut microbiota and microbiome during both NC and HFHS diets.

### GBP induces specific changes in gut microbiota and microbiome of NC- and HFHS-fed WT mice

To specifically determine the role of diet in relation to WT genotype, we compared gut microbiota and microbiome of all of the WT mice fed either a NC or a HFHS diet in this work. All groups of mice displayed at least a specific microbial taxon (Supplementary Fig. 8A). In detail, on NC, the phyla *Bacteroidetes* and *Actinobacteria* had a significantly higher abundance in laparotomized mice, while bacteria from order *Lactobacilalles* showed a significantly higher abundance in GBP mice. On HFHS diet, the phylum *Proteobacteria* together with bacteria from genus *Bacteroides* had a significantly higher abundance in GBP mice, whereas laparotomized mice had a significantly higher abundance of *Firmicutes* and PF laparotomized HFHS-fed mice had a significantly higher abundance of family *Rikenellaceae* and genus *Bilophila*. The overall diversity of gut microbiota of all groups of mice was highly and significantly divergent, mostly based on Chao-1 and, to a lesser extent, to Fisher-alpha indices (Supplementary Fig. 8B). The overall profile of gut microbiota of all groups of mice was also significantly diverse, with NC-fed laparotomized mice being the most divergent group compared to HFHS-fed mice (Supplementary Fig. 8C). Consistently, all groups of WT mice displayed microbial inferred functions enriched by GBP and diet (Supplementary Fig. 8D). The profile of gut microbiome of all groups of mice was also significantly diverse, with HFHS-fed GBP mice being the most diverse group when compared to HFHS-fed mice (Supplementary Fig. 8E).

### GBP induces specific changes in gut microbiota and microbiome of NC- and HFHS-fed iG6PC-KO mice

To specifically determine the role of diet in relation to iG6PC-KO genotype, we compared the gut microbiota and microbiome of all of the iG6PC-KO mice fed either a NC or a HFHS diet in this work. All groups of mice displayed specific taxa of gut microbiota (Supplementary Fig. 9A). In detail, on a NC, the class of *Bacilli* had a significantly higher abundance in laparotomized iG6PC-KO mice whereas the gut microbiota of GBP iG6PC-KO mice showed significantly higher abundance of phyla *Proteobacteria*, *Bacteroidetes* and *Actinobacteria*. On a HFHS feeding, bacteria from order *Enterobacteriales* and genus *Roseburia* showed a significantly higher abundance in GBP iG6PC-KO mice; genus *Anaerovorax* had a significantly higher abundance in laparotomized iG6PC-KO mice whereas the gut microbiota of PF iG6PC-KO mice was characterized by a significantly higher abundance of bacteria from *Christensenellaceae* family and genus *Alistipes*. The overall diversity of gut microbiota of all of the groups of mice was highly and significantly different, based on both Chao-1 and Fisher-alpha indices (Supplementary Fig. 9B). The overall profile of gut microbiota of all groups of mice was significantly diverse, with a more pronounced diet effect (Supplementary Fig. 9C), compared to WT mice (Supplementary Fig. 9C). These data suggest a synergic effect by GBP and diet on gut microbiota in iG6PC-KO mice. Regarding gut microbiome, all groups of iG6PC-KO mice displayed specific microbial inferred functions enriched by GBP and diet (Supplementary Fig. 9D) and a significantly diverse overall profile (Supplementary Fig. 9E).

Finally, it is noteworthy that the comparative analysis of both gut microbiota (Supplementary Fig. 10) and microbiome (Supplementary Fig. 11) could identify at least a specific microbial taxon and microbial inferred functions enriched in each of the ten groups of mice in this study. Only the gut microbiota of HFHS-fed iG6PC-KO GBP mice did not significantly vary compared to all the other groups of mice (since it does not appear in Supplementary Fig. 10) whereas its microbiome showed specific enriched inferred functions Supplementary Fig. 11. Overall, these data show that GBP induces highly specific changes in both gut microbiota and microbiome in both WT and iG6PC-KO mice, regardless of diet.

**Figure 10:**
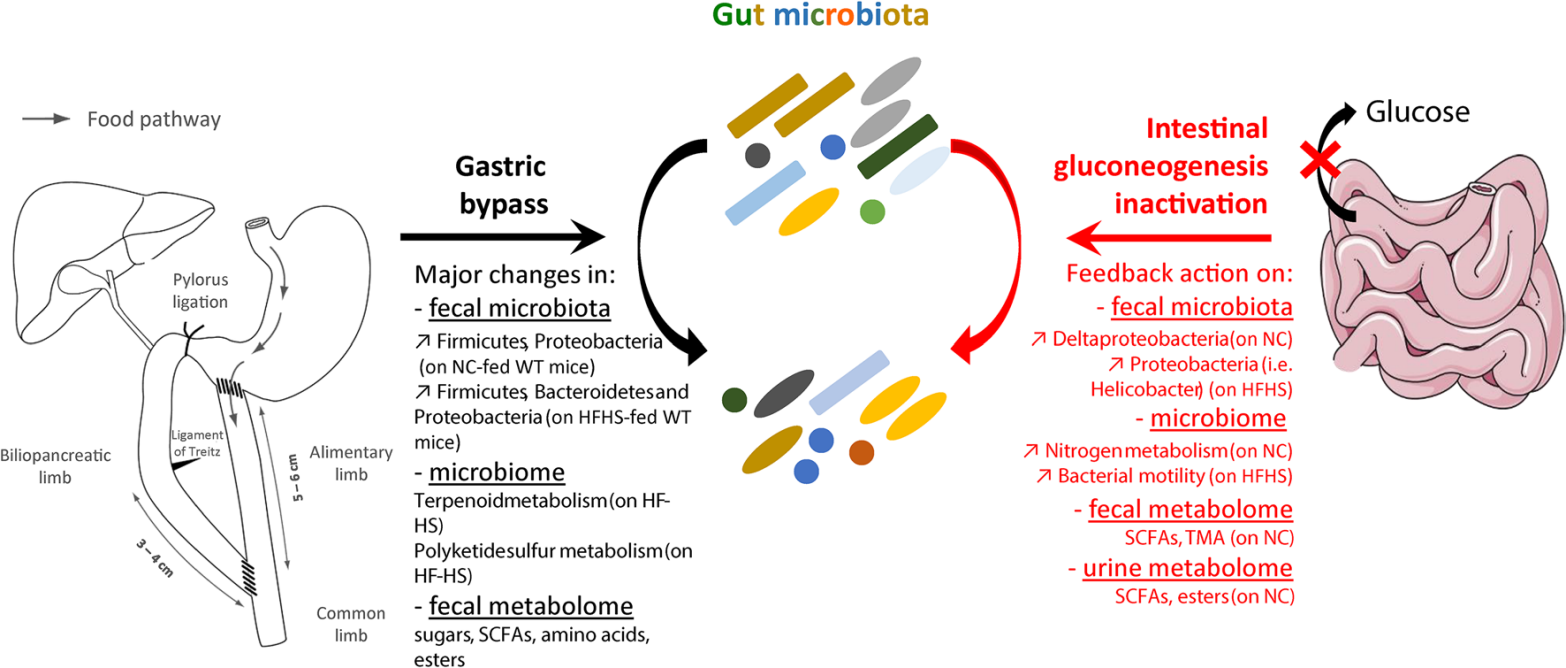
Graphical summary of the effects reported in this study for both intestinal gluconeogenesis inactivation and gastric bypass on gut microbiota. Original gastric bypass picture published in 10.1038/srep44856. Small intestine image from https://smart.servier.com/.

The major changes induced by both gastric bypass and intestinal glucose inactivation on gut microbiota, microbiome as well as fecal and urine metabolome are summarized in Fig. 10.

## Discussion

In this study, we evaluated separately and combinedly the effects of both IGN inactivation and GBP on gut microbiota and microbiome in NC- and HFHS-fed mice. Our data highlight that both IGN and GBP have the capacity to modulate gut microbiota and microbiome regardless of the two diets. We hypothesized that IGN inactivation might change the content of SCFAs in the lumen, leading to a modification in bacterial cross-feeding and a final shaping of gut microbiota. Consistently with our hypothesis, we found a reduction in the amount of fecal acetate, which is the major SCFA produced. In accordance with our data, IGN inactivation in HFHS-fed mice supplemented with fructooligosaccharides also decreased cecal acetate content without measurable changes in propionate and butyrate^12^. We also showed that IGN inactivation on NC induced a dysbiosis with significantly higher relative abundance of bacteria belonging to major phyla such as *Firmicutes* (significantly higher relative abundance of genus *CandidatusArthomitus* and family *LachnospiraceaeNK4A136*), *Proteobacteria* (significantly higher relative abundance of family *Desulfovibrionaceae* and genus *Desulfovibrio*) and *Bacteroidetes* (significantly higher relative abundance of genera *Odoribacter, Alistipes* and *Rikenella*).

In line with our previous report, during HFHS-feeding, mice with IGN inactivation displayed higher abundance of *Firmicutes*^11^. HFHS-fed mice with IGN inactivation also had a significant higher abundance of *Proteobacteria* such as *Helicobacter*, which was not observed during HFHS feeding or IGN inactivation alone. This evidence may suggest a detrimental effect of the suppression of IGN on gut microbial community. In fact, during NC feeding, WT mice compared to iG6PC-KO mice had a significant higher abundance of genus *Parabacteroides*, shown to alleviate obesity and metabolic alterations in both ob/ob and HFD-fed mice, by activating IGN via the production of succinate^24^. Additionally, once combined with a nutritional challenge such as a HFHS diet, IGN inactivation may be deleterious for the host via the subsequent increase of pathogens such as *Helicobacter*^25,26^. This interpretation is corroborated by the significant higher bacterial motility-related inferred functions observed in the gut microbiome of HFHS-fed iG6PC-KO mice, the efficacy of bacterial motility being a distinguishing trait of pathogens^27^. Further analyses including multiple time-points of fecal sampling during HFHS diet should improve our knowledge on the causality relationship of the diet for the changes in gut microbiota composition.

GBP induced significantly higher abundance of *Firmicutes* and *Proteobacteria* on NC (limited sample size due to complexity of GBP surgery may have underpowered the experimental comparison between NC_WT_Lap and NC_WT_GBP groups) and significantly higher abundance of *Firmicutes*, *Bacteroidetes* and *Proteobacteria* on HFHS diet. *Firmicutes* are major producers of SCFAs such as butyrate. Therefore, a shift in gut microbiota towards SCFAs-producing bacteria could account, at least in part, for the metabolic benefits of GBP, since SCFAs induce multiple metabolic benefits for the host^28^ including the activation of IGN^11–13^. We did not observe changes in fecal butyrate content regardless of genotype, GBP or diet in our study. Indeed, changes in the amount of propionate and butyrate are more difficult to highlight in feces since they are mainly absorbed by colonocytes^29,30^. A similarity between the shift in gut microbiota induced by IGN inactivation and GBP was observed at the level of phyla (*Firmicutes, Bacteroidetes* and *Proteobacteria*). However, at sublevels such as family, genus and species the two shifts showed some differences, underlining the impact of both IGN inactivation and GBP on NC and HFHS.

More importantly, IGN but not GBP induced changes in urine acetate levels. Butyrate is the preferred energy substrate for colonocytes, while acetate crosses gut barrier to reach the portal vein. Propionate is utilised by the intestine and the liver. This is not the case of acetate. As a result, acetate is the most abundant SCFA in the systemic circulation whereas only small amounts of butyrate and propionate could be found in periphery^31^. Microbiota shaping by IGN inactivation might thus control systemic acetate plasma levels and consequently negatively impact on host metabolism. Overall, the detrimental effects deriving from IGN inactivation agree with the previous observation that SCFAs activate IGN^28^ thus leading to huge metabolic benefits.

It is noteworthy that GBP counteracted changes induced by IGN inactivation on gut microbiota and microbiome during both NC and HFHS diet. On one hand, we observed that NC-fed iG6PC-KO mice that underwent GBP had higher bifidobacteria at the taxonomic levels of order, family and genus. Bifidobacteria are known probiotics^32^, therefore the combination of GBP and IGN inactivation during NC feeding appears to benefit the host by shifting gut microbiota towards an enrichment in beneficial microbes. By contrast, on a HFHS feeding, the combination of GBP and IGN inactivation appears to disadvantage the host, by shifting gut microbiota towards an enrichment in detrimental microbes, such as those from genus *Escherichia*_*Shigella*, which are known enterobacteria pathogens^33^. During HFHS feeding, GBP induced a significant decrease in Fisher and Chao-1 diversity indices in both WT and iG6PC-KO mice. These indices are related to rare microbial taxa, suggesting that this reduction is not affecting the overall gut microbiota diversity.

The family *Desulfovibrionaceae* was significantly and positively correlated with the indole alkaloid biosynthesis microbial pathway, when combining microbiota and microbiome in both all WT and iG6PC-KO mice in this study. A vast body of publications points out the role of indole as a microbial metabolite responsible for many regulatory effects on host functions, such as inflammation and gut barrier^28^. The *Desulfovibrionaceae* family showed a significant higher relative abundance in the gut microbiota of both NC- and HFHS-fed WT mice that underwent GBP. This evidence appears in keeping with the benefits of GBP on the detrimental changes induced on gut microbiota either by IGN inactivation on NC or by a HFHS diet. However, the relative abundance of *Desulfovibrionaceae* family was also significantly higher in gut microbiota of NC-fed iG6PC-KO mice. This evidence suggests that the presence of *Desulfovibrionaceae* family may be necessary but not sufficient to explain alone the beneficial effects of GBP to the host. Rather, the overall GBP-modified gut microbiota would account for these positive effects.

When considering the impact on gut microbiota of both IGN inactivation and GBP during NC and HFHS feeding, the most striking result is the fact that every group of mice, regardless of genotype and diet, had at least a specific taxon and/or a microbial function enriched. This evidence underlines deep modifications induced by IGN inactivation and GBP on gut microbiota. It must be mentioned that the changes in microbiota induced by the diet were studied through the comparison of NC to HFHS fed mice. However, the specific effects of the HFHS diet on microbiota and microbiome might be improved by further analyses including multiple time-points of fecal sampling to decipher to which extent any diet account for the observed changes.

By showing that GBP may also counteract, at least in part, IGN inactivation-induced gut microbiota dysbiosis, our study also provides a new rationale susceptible to account for the metabolic benefits of GBP. Finally, the inactivation of IGN reduces the intestinal capacity to metabolize specific bacterial metabolites such as SCFAs, changing both ecological structure and functions of the gut microbiota.

## Materials and methods

### Animal models

All experiments were performed according to the principles and guidelines established by the European Convention for the Protection of Laboratory Animals. Protocols were approved by our regional animal care committee (C2EA-55, Université Lyon 1, Lyon) and by the Ministry of Higher Education and Research (Agreement project number: Apafis#11929–2017102421331413 v1). Male C57Bl/6 J wild-type mice (WT) were purchased from Charles River Laboratories at 4 weeks of age. Male iG6PC-KO mice, with an intestine-specific disruption of the catalytic subunit (*G6pc1*) of glucose-6 phosphatase, the key enzyme in endogenous glucose production, were generated as described previously^34^. Briefly, 7–8-wk-old iG6PC-KO mice received a daily 100 µL injection of Tamoxifen (10 mg/mL, Sigma) on 5 consecutive days. WT mice did not receive tamoxifen as they will undergo major surgery and a HFHS diet for 20 weeks (see below). In fact, the putative metabolic effect of tamoxifen in adult mice decreases sharply after 5 weeks, thus its effect will likely be dominated by nutritional and/or surgical cues^35,36^.

All mice were housed in the animal facility of Lyon 1 University under controlled temperature (22 ± 2 °C) and lighting (12 h light/dark cycle with light at 7 a.m.) with free access to food and water.

To induce obesity, 4-weeks old WT and iG6PC-KO mice were placed on high-fat/high-sucrose (HFHS) diet for 20 weeks prior to surgery. HFHS diet, consisting of 36.1% fat, 35% carbohydrates (50% maltodextrine + 50% sucrose) and 19.8% proteins, was produced by the Unité de Préparation des Aliments Expérimentaux (UE SAAJ INRAE, Jouy-en-Josas, France). For experiments in lean animals, WT and iG6PC-KO mice were maintained on standard diet (SAFE A04, Augis, France) and surgery was performed at 24-weeks old. All animals were maintained on their respective diet after surgery.

The duodenal-jejunal bypass surgery (GBP) was performed as previously described in 24-weeks old mice^37^. For each genotype (WT and iG6PC-KO), GBP (duodenal-jejunal)-operated mice and sham-operated mice groups were constituted at the day of surgery; sham operation consisting in a laparotomy^37^. Since GBP mice fed a HFHS diet ate less during the first ten days after the operation, experiments on HFHS fed mice involved a third group of sham-operated PF mice (sham-PF) mice^37^. Twenty-two days after surgery, mice were placed fro 48 h in a metabolic cage for collecting urine and feces.

Twenty-five days after surgery, mice were fasted for 6 h and next killed by cervical dislocation. The caecum content was sampled and immediately frozen at − 80 °C for further analyses. All groups co-housed almost 6 months (20 weeks plus 25 days) since the beginning of the diet, which equilibrates gut microbiota of mice before any intervention^38^. Mice were randomly associated to control or any other treatment (HF/HS and/or surgery) group. Given the murine models used in this work, researchers were aware of the group allocation during all the study. ARRIVE guidelines were followed. “n” = 35 for WT mice (NC_WT_Lap = 4; NC_WT_GBP = 3; HFHS_WT_Lap = 9; HFHS_WT_Lap_PF = 10; HFHS_WT_GBP = 9); “n” = 26 for KO mice (NC_iG6PC_KO_Lap = 5; NC_iG6PC_KO_GBP = 4; HFHS_iG6PC_KO_Lap = 6; HFHS_iG6PC_KO_Lap_PF = 6; HFHS_iG6PC_KO_GBP = 5). Exclusion criteria of mice were only based on the success of surgery and on the quality and quantity of the collected urine and feces samples.

### Taxonomic and predicted functional analysis of gut microbiota

Total DNA was extracted from caecum content at VAIOMER SAS (https://www.vaiomer.com/, Toulouse, France). The 16S bacterial DNA V3-V4 regions were targeted by using Vaiomer universal 16S primers and analysed by MiSeq kit V3 50,000 raw read pairs per sample, which was experimentally determined to be the number of reads to have exhaustive coverage of the community profiles present in high diversity samples. The cladograms in Figs. 1A,E, 4A, 6A, 7A, 9A, Supplementary Fig. 8A, Supplementary Fig. 9A as well as LDA scores in Figs. 1D, 1H, 4D, 6D, 7D, 9D, Supplementary Fig. 8D, Supplementary Fig. 9D and Supplementary Figs. 10–11 were drawn using the Huttenhower Galaxy web application (https://huttenhower.sph.harvard.edu/galaxy) via the LEfSe algorithm (Galaxy Version 1.0)^39^. Briefly, P values were calculated based on an alpha value for the factorial Kruskal–Wallis test among classes an alpha value for the pairwise Wilcoxon test between subclasses (both set at 0.05 or 0.01 as reported in figures) and a threshold on the logarithmic LDA score for discriminative features, set to 2.0 or as reported in figure legends. Diversity indices were calculated using the software PAST 4 (Hammer, Ø., Harper, D.A.T., and P. D. Ryan, 2001. PAST: Paleontological Statistics Software Package for Education and Data Analysis. Palaeontologia Electronica 4(1): 9 pp). The mean of each diversity index can be found at the following link https://www.nhm.uio.no/english/research/infrastructure/past/help/diversity.html.

The predictive functional analysis (gut microbiome) of gut microbiota was performed via PICRUSt^40^. Original sources of method description as already published^41–43^.

### Metabolomic analysis

*Sample preparation*. Fecal samples (100 mg) were homogenized using the FastPrep-24 (MP Biomedicals, Irvine, CA, USA) homogenizer in 0.5 ml of phosphate buffer (0.2 M, pH = 7) prepared in deuterium oxide (D_2_O) and containing 1 mM trimethylsilylpropionic acid (TSP). Samples were left on ice for 1 min and homogenized again. Samples were then centrifuged (10000*g*, 10 min, 4 °C) and supernatants were collected. The remaining pellet was further extracted as described above. Supernatants obtained from two runs of extraction were combined and centrifuged at 10,000*g* for 10 min at 4 °C. A total of 600 µL of supernatant was transferred into 5 mm NMR tubes. As for urines, they were collected over 48 h on metabolic cages. Prior to analysis, urine samples were thawed at room temperature. Then, 200 µL of phosphate buffer (0.2 M, pH = 7) prepared in D_2_O and containing 1 mM TSP was added to 500 µL of urine sample. The mixture was vortexed, centrifuged (5500*g*, 15 min, 4 °C), and 600 µL of supernatant were transferred to a 5 mm NMR tube.

^*1*^*H NMR acquisition.* ^1^H NMR spectra were obtained at 300 K, on a Bruker Avance III HD 600 MHz NMR spectrometer (Bruker Biospin, Rheinstetten, Germany) operating at 600.13 MHz for proton frequency using an inverse detection 5 mm ^1^H-^13^C-^15^ N-^31^P cryoprobe. ^1^H NMR spectra of urine samples were acquired using the « noesypr1d » (Bruker Library) pulse sequence with water suppression during the relaxation delay (5 s) and mixing time (100 ms). A total of 256 transients were collected into 65,536 data points using a spectral width of 20 ppm and an acquisition time of 2.7 s. ^1^H NMR spectra of fecal samples were acquired using the Carr-Purcell-Meiboom-Gill (CPMG) spin echo pulse sequence with presaturation, with a total spin-echo delay of 320 ms to attenuate broad signals from macromolecules. A total of 256 transients were collected into 65,536 data points using a spectral width of 20 ppm, a relaxation delay of 5 s and an acquisition time of 2.7 s. Prior to Fourier transformation, an exponential line broadening function of 0.3 Hz was applied to the Free Induction Decays (FID). All NMR spectra were phase- and baseline-corrected and referenced to the chemical shift of TSP (0 ppm) using Topspin (V3.2, Bruker Biospin, Germany). Original sources of method description as already published^41–43^.

### Statistical analysis

Data for diversity indices and metabolome analyses are presented as mean ± SD. Statistical analyses were performed by 2-way ANOVA followed by a 2-stage linear step-up procedure of Benjamini, Krieger and Yekutieli to correct for multiple comparisons by controlling the False Discovery Rate (< 0.05), by using GraphPad Prism version 9.3.0 for Windows Vista (www.graphpad.com, GraphPad Software, San Diego, CA). Values were considered significant starting at *P* < 0.05 or as reported. For the taxonomical and predictive functional analysis of gut microbiota significant values were considered starting at *P* < 0.05 or as reported. Principal Component Analysis graphs were drawn and related statistical analyses were performed using PAST 4 0.08 (https://www.nhm.uio.no/english/research/infrastructure/past/) and calculating Bray–Curtis distance with 1-way PERMANOVA analysis with Bonferroni’s correction. Heat-maps based on Pearson distance and a complete linkage were drawn by using PermutMatrix 1.9.4 (http://www.atgc-montpellier.fr/permutmatrix/)^44^. Original sources of method description as already published^41–43^.

## Supplementary Information

Supplementary Legends.

Supplementary Figures.

## Data Availability

The microbiota datasets generated and analysed in the current study are available in the Sequence Read Archive (SRA) repository (https://submit.ncbi.nlm.nih.gov/subs/sra/) with the assigned identifier PRJNA595458. All metabolomics data are within the manuscript.

## Abbreviations

ANOVA: Analysis of variance
GBP: Gastric bypass
HFHS: High-fat high-sucrose
IGN: Intestinal glucose production
iG6PC-KO: Intestinal glucose-6-phosphatase catalytic subunit knock-out
Lap: Laparotomized
LDA: Linear discriminant analysis
NC: Normal chow
PF: Pair-fed
SCFAs: Short chain fatty acids
T2DM: Type 2 diabetes mellitus
TMA: Trimethylamine
WT: Wild-type

## References

[1] Debedat J, Sokolovska N, Coupaye M, Panunzi S, Chakaroun R, Genser L, de Turenne G, Bouillot JL, Poitou C, Oppert JM, Bluher M, Stumvoll M, Mingrone G, Ledoux S, Zucker JD, Clement K, Aron-Wisnewsky J (2018). Long-term relapse of type 2 diabetes after Roux-en-Y gastric bypass: Prediction and clinical relevance. Diabetes Care.

[2] Diedisheim M, Poitou C, Genser L, Amouyal C, Bouillot JL, Ciangura C, Oppert JM, Clement K, Aron-Wisnewsky J (2021). Weight loss after sleeve gastrectomy: Does type 2 diabetes status impact weight and body composition trajectories?. Obes. Surg..

[3] Silveira FC, Docherty NG, Sallet PC, Moraes M, Monclaro T, Arruda ESM, Pizani CE, Sallet JA, le Roux CW (2020). Early post-operative weight change after Roux-en-Y gastric bypass predicts weight loss at 12-month follow-up. Obes. Surg..

[4] Lassailly G, Caiazzo R, Ntandja-Wandji LC, Gnemmi V, Baud G, Verkindt H, Ningarhari M, Louvet A, Leteurtre E, Raverdy V, Dharancy S, Pattou F, Mathurin P (2020). Bariatric surgery provides long-term resolution of nonalcoholic steatohepatitis and regression of fibrosis. Gastroenterology.

[5] Mithieux G (2018). Gut microbiota and host metabolism: What relationship. Neuroendocrinology.

[6] Tremaroli V, Karlsson F, Werling M, Stahlman M, Kovatcheva-Datchary P, Olbers T, Fandriks L, le Roux CW, Nielsen J, Backhed F (2015). Roux-en-Y gastric bypass and vertical banded gastroplasty induce long-term changes on the human gut microbiome contributing to fat mass regulation. Cell Metab...

[7] Kim M, Son YG, Kang YN, Ha TK, Ha E (2015). Changes in glucose transporters, gluconeogenesis, and circadian clock after duodenal-jejunal bypass surgery. Obes. Surg..

[8] Sun D, Wang K, Yan Z, Zhang G, Liu S, Liu F, Hu C, Hu S (2013). Duodenal-jejunal bypass surgery up-regulates the expression of the hepatic insulin signaling proteins and the key regulatory enzymes of intestinal gluconeogenesis in diabetic Goto-Kakizaki rats. Obes. Surg..

[9] Troy S, Soty M, Ribeiro L, Laval L, Migrenne S, Fioramonti X, Pillot B, Fauveau V, Aubert R, Viollet B, Foretz M, Leclerc J, Duchampt A, Zitoun C, Thorens B, Magnan C, Mithieux G, Andreelli F (2008). Intestinal gluconeogenesis is a key factor for early metabolic changes after gastric bypass but not after gastric lap-band in mice. Cell Metab...

[10] Yan Y, Zhou Z, Kong F, Feng S, Li X, Sha Y, Zhang G, Liu H, Zhang H, Wang S, Hu C, Zhang X (2016). Roux-en-Y gastric bypass surgery suppresses hepatic gluconeogenesis and increases intestinal gluconeogenesis in a T2DM rat model. Obes. Surg..

[11] De Vadder F, Kovatcheva-Datchary P, Goncalves D, Vinera J, Zitoun C, Duchampt A, Backhed F, Mithieux G (2014). Microbiota-generated metabolites promote metabolic benefits via gut-brain neural circuits. Cell.

[12] De Vadder F, Kovatcheva-Datchary P, Zitoun C, Duchampt A, Backhed F, Mithieux G (2016). Microbiota-produced succinate improves glucose homeostasis via intestinal gluconeogenesis. Cell Metab..

[13] Soty M, Gautier-Stein A, Rajas F, Mithieux G (2017). Gut-brain glucose signaling in energy homeostasis. Cell Metab..

[14] Hayes MT, Foo J, Besic V, Tychinskaya Y, Stubbs RS (2011). Is intestinal gluconeogenesis a key factor in the early changes in glucose homeostasis following gastric bypass?. Obes. Surg..

[15] Mithieux, G. Comment about intestinal gluconeogenesis after gastric bypass in human in relation with the paper by Hayes et al., Obes. Surg. 2011. Obes. Surg. **22**(12), 1920–1922; author reply 3–4 (2012). 10.1007/s11695-012-0755-4 22961684

[16] Gutierrez-Repiso C, Garcia-Serrano S, Moreno-Ruiz FJ, Alcain-Martinez G, Rodriguez-Pacheco F, Garcia-Fuentes E (2017). Jejunal gluconeogenesis associated with insulin resistance level and its evolution after Roux-en-Y gastric bypass. Surg. Obes. Relat. Dis..

[17] Swanson KS, de Vos WM, Martens EC, Gilbert JA, Menon RS, Soto-Vaca A, Hautvast J, Meyer PD, Borewicz K, Vaughan EE, Slavin JL (2020). Effect of fructans, prebiotics and fibres on the human gut microbiome assessed by 16S rRNA-based approaches: A review. Benef. Microbes.

[18] David LA, Maurice CF, Carmody RN, Gootenberg DB, Button JE, Wolfe BE, Ling AV, Devlin AS, Varma Y, Fischbach MA, Biddinger SB, Dutton RJ, Turnbaugh PJ (2014). Diet rapidly and reproducibly alters the human gut microbiome. Nature.

[19] Carmody RN, Gerber GK, Luevano JM, Gatti DM, Somes L, Svenson KL, Turnbaugh PJ (2015). Diet dominates host genotype in shaping the murine gut microbiota. Cell Host Microbe.

[20] Liu W, Zassoko R, Mele T, Luke P, Sun H, Liu W, Garcia B, Jiang J, Wang H (2008). Establishment of duodenojejunal bypass surgery in mice: A model designed for diabetic research. Microsurgery.

[21] Woods M, Lan Z, Li J, Wheeler MB, Wang H, Wang R (2011). Antidiabetic effects of duodenojejunal bypass in an experimental model of diabetes induced by a high-fat diet. Br. J. Surg..

[22] Boland B (2019). The PYY/Y2R-deficient mouse responds normally to high-fat diet and gastric bypass surgery. Nutrients.

[23] Meirelles K, Ahmed T, Culnan DM, Lynch CJ, Lang CH, Cooney RN (2009). Mechanisms of glucose homeostasis after Roux-en-Y gastric bypass surgery in the obese, insulin-resistant Zucker rat. Ann. Surg..

[24] Wang K, Liao M, Zhou N, Bao L, Ma K, Zheng Z, Wang Y, Liu C, Wang W, Wang J, Liu SJ, Liu H (2019). Parabacteroides distasonis alleviates obesity and metabolic dysfunctions via production of succinate and secondary bile acids. Cell. Rep..

[25] Alvarado CG, Kocsis AG, Hart ML, Crim MJ, Myles MH, Franklin CL (2015). Pathogenicity of Helicobacter ganmani in mice susceptible and resistant to infection with *H. hepaticus*. Comp. Med..

[26] Smet A, Menard A (2020). Review: Other helicobacter species. Helicobacter.

[27] Chaban B, Hughes HV, Beeby M (2015). The flagellum in bacterial pathogens: For motility and a whole lot more. Semin. Cell. Dev. Biol..

[28] Abdul Rahim MBH, Chilloux J, Martinez-Gili L, Neves AL, Myridakis A, Gooderham N, Dumas ME (2019). Diet-induced metabolic changes of the human gut microbiome: Importance of short-chain fatty acids, methylamines and indoles. Acta Diabetol..

[29] Rechkemmer G, Ronnau K, von Engelhardt W (1988). Fermentation of polysaccharides and absorption of short chain fatty acids in the mammalian hindgut. Comp. Biochem. Physiol. A Comp. Physiol..

[30] Ruppin H, Bar-Meir S, Soergel KH, Wood CM, Schmitt MG (1980). Absorption of short-chain fatty acids by the colon. Gastroenterology.

[31] Koh A, De Vadder F, Kovatcheva-Datchary P, Backhed F (2016). From dietary fiber to host physiology: Short-chain fatty acids as key bacterial metabolites. Cell.

[32] Gibson GR, Hutkins R, Sanders ME, Prescott SL, Reimer RA, Salminen SJ, Scott K, Stanton C, Swanson KS, Cani PD, Verbeke K, Reid G (2017). Expert consensus document: The International Scientific Association for Probiotics and Prebiotics (ISAPP) consensus statement on the definition and scope of prebiotics. Nat. Rev. Gastroenterol. Hepatol..

[33] Serino M (2018). Molecular paths linking metabolic diseases, gut microbiota dysbiosis and enterobacteria infections. J. Mol. Biol..

[34] Penhoat A, Mutel E, Amigo-Correig M, Pillot B, Stefanutti A, Rajas F, Mithieux G (2011). Protein-induced satiety is abolished in the absence of intestinal gluconeogenesis. Physiol. Behav..

[35] Hesselbarth N, Pettinelli C, Gericke M, Berger C, Kunath A, Stumvoll M, Bluher M, Kloting N (2015). Tamoxifen affects glucose and lipid metabolism parameters, causes browning of subcutaneous adipose tissue and transient body composition changes in C57BL/6NTac mice. Biochem. Biophys. Res. Commun..

[36] Ye R, Wang QA, Tao C, Vishvanath L, Shao M, McDonald JG, Gupta RK, Scherer PE (2015). Impact of tamoxifen on adipocyte lineage tracing: Inducer of adipogenesis and prolonged nuclear translocation of Cre recombinase. Mol. Metab..

[37] Barataud A, Goncalves D, Vinera J, Zitoun C, Duchampt A, Gautier-Stein A, Mithieux G (2017). Absence of role of dietary protein sensing in the metabolic benefits of Duodenal–Jejunal bypass in the mouse. Sci. Rep..

[38] Laukens D, Brinkman BM, Raes J, De Vos M, Vandenabeele P (2016). Heterogeneity of the gut microbiome in mice: Guidelines for optimizing experimental design. FEMS Microbiol. Rev..

[39] Segata N, Izard J, Waldron L, Gevers D, Miropolsky L, Garrett WS, Huttenhower C (2011). Metagenomic biomarker discovery and explanation. Genome Biol..

[40] Langille MG, Zaneveld J, Caporaso JG, McDonald D, Knights D, Reyes JA, Clemente JC, Burkepile DE, Vega Thurber RL, Knight R, Beiko RG, Huttenhower C (2013). Predictive functional profiling of microbial communities using 16S rRNA marker gene sequences. Nat. Biotechnol..

[41] Brehin C, Dubois D, Dicky O, Breinig S, Oswald E, Serino M (2020). Evolution of gut microbiome and metabolome in suspected necrotizing enterocolitis: A case-control study. J. Clin. Med..

[42] Brochot A, Azalbert V, Landrier JF, Tourniaire F, Serino M (2019). A two-week treatment with plant extracts changes gut microbiota, caecum metabolome, and markers of lipid metabolism in ob/ob mice. Mol. Nutr. Food Res..

[43] Nicolas S, Blasco-Baque V, Fournel A, Gilleron J, Klopp P, Waget A, Ceppo F, Marlin A, Padmanabhan R, Iacovoni JS, Terce F, Cani PD, Tanti JF, Burcelin R, Knauf C, Cormont M, Serino M (2017). Transfer of dysbiotic gut microbiota has beneficial effects on host liver metabolism. Mol. Syst. Biol..

[44] Caraux G, Pinloche S (2005). PermutMatrix: A graphical environment to arrange gene expression profiles in optimal linear order. Bioinformatics.

