# Supplementary material for: Intestinal gluconeogenesis shapes gut microbiota, fecal and urine metabolome in mice with gastric bypass surgery
Source: Sci Rep. 2022 Jan 26;12:1415. doi: 10.1038/s41598-022-04902-y (PMC8791999; doi:10.1038/s41598-022-04902-y)
Supplement: Supplementary file 1 — Supplementary Legends. [file 41598_2022_4902_MOESM1_ESM.docx]

**Intestinal Gluconeogenesis Shapes Gut Microbiota, Fecal and Urine Metabolome in Mice with Gastric Bypass Surgery**

**Justine Vily-Petit^1^, Aude Barataud^1^, Carine Zitoun^1^,**

**Amandine Gautier-Stein^1^, Matteo Serino^2*$^ & Gilles Mithieux^1*$^**

^1^Université Claude Bernard Lyon 1, Université de Lyon, INSERM UMR-S1213, Lyon, France. ^2^IRSD, Université de Toulouse, INSERM, INRAE, ENVT, UPS, Toulouse, France.

*Corresponding authors: [gilles.mithieux@univ-lyon1.fr](mailto:gilles.mithieux@univ-lyon1.fr), <https://orcid.org/0000-0003-3579-8529>. Tel : +33 4 78 77 87 88 ; [matteo.serino@inserm.fr](mailto:matteo.serino@inserm.fr); <https://orcid.org/0000-0003-4644-8532>; Tel: +33 5 62 74 45 25. ^$^Equal contribution.

**Short title:** gastric bypass and gut gluconeogenesis

**Supplementary Figure legends**

**Supplementary Figure 1. Metabolomic analysis in feces from NC-fed WT and intestinal Glucose-6-Phosphatase C KO mice**. PCA of fecal metabolome: A) sugars, B) amino acids, C) esters and other metabolites. For hystograms (A-C), **P<0.01, ***P<0.001, ****P<0.0001, 2-way ANOVA followed by a 2-stage linear step-up procedure of Benjamini, Krieger and Yekutieli to correct for multiple comparisons by controlling the False Discovery Rate (<0.05). “n” for: NC_WT_Lap = 4, NC_iG6PC_KO_Lap = 5.

**Supplementary Figure 2. Metabolomic analysis in urine from NC-fed WT and intestinal Glucose-6-Phosphatase C KO mice**. A) PCA of TMA and other related metabolites in urine metabolome. B) urine volume collected over 24 and 48 hours. C) food intake measurement. D) body weight. **P<0.01, 2-way ANOVA followed by a 2-stage linear step-up procedure of Benjamini, Krieger and Yekutieli to correct for multiple comparisons by controlling the False Discovery Rate (<0.05). “n” for: NC_WT_Lap = 3 (4 for B,C,D), NC_iG6PC_KO_Lap = 5.

**Supplementary Figure 3. Metabolomic analysis in feces and urine from NC-fed WT mice following gastric bypass.** PCA of: A) TMA and other related metabolites from fecal metabolome, B) overall urine metabolome, C) TMA and other related metabolites, D) SCFAs, E) esters and other metabolites, F) glycine and other metabolites. G) Urine volume collected over 24h and 48h. **P<0.01, 2-way ANOVA followed by a 2-stage linear step-up procedure of Benjamini, Krieger and Yekutieli to correct for multiple comparisons by controlling the False Discovery Rate (<0.05). “n” for: NC_WT_Lap = 3 (4 for G), NC_WT_GBP = 3.

**Supplementary Figure 4. Metabolic parameters in NC-fed WT and intestinal Glucose-6-Phosphatase C KO mice following gastric bypass over 24 and 48 hours.** A) Food intake measurement. B) body weight. “n” for: NC_WT_GBP = 3, NC_iG6PC_KO_GBP = 4.

**Supplementary Figure 5. Metabolomic analysis in feces from NC-fed intestinal Glucose-6-Phosphatase C KO mice following gastric bypass.** PCA of: A) overall fecal metabolome, B) sugars, C) esters and other metabolites, D) SCFAs, E) TMA and other related metabolites, F) amino acids. ***P<0.001, 2-way ANOVA followed by a 2-stage linear step-up procedure of Benjamini, Krieger and Yekutieli to correct for multiple comparisons by controlling the False Discovery Rate (<0.05). “n” for: NC_iG6PC_KO_Lap = 5, NC_iG6PC_KO_GBP = 4.

**Supplementary Figure 6. Metabolomic analysis in urine from NC-fed intestinal Glucose-6-Phosphatase C KO mice following gastric bypass.** PCA of: A) TMA and other related metabolites, B) SCFAs, C) esters and other metabolites. D) urine volume collected over 24h and 48h. **P<0.01, ***P<0.001, 2-way ANOVA followed by a 2-stage linear step-up procedure of Benjamini, Krieger and Yekutieli to correct for multiple comparisons by controlling the False Discovery Rate (<0.05). “n” for: NC_iG6PC_KO_Lap = 5, NC_iG6PC_KO_GBP = 4.

**Supplementary Figure 7. Correlation between gut (caecum) microbiota, microbiome, fecal or urine metabolome in NC-fed WT and intestinal Glucose-6-Phosphatase C KO mice which underwent gastric bypass surgery**. A) Heatmap between all identified bacterial groups (591), microbial inferred functions (328) and feces metabolites (61): top dendrogram shows clusters of mice; left dendogram shows clusters of parameters; values have been mean centred and divided by standard deviation. B) Heatmap between all identified bacterial groups (591), microbial inferred functions (328) and urine metabolites (36): top dendrogram shows clusters of mice; left dendogram shows clusters of parameters; values have been mean centred and divided by standard deviation. C) cluster from heatmap in B between a bacterial taxon (B39), two microbial functions (F276, F297) and a urine metabolite (UriMet598), identified in the below reported table. (C) correlations between parameters cited above. “n” for: NC_WT_Lap = 3 (4 for A); NC_WT_GBP = 3; NC_iG6PC_KO_Lap = 5, NC_iG6PC_KO_GBP = 4.

**Supplementary Figure 8. Gastric bypass changes gut (caecum) microbiota and microbiome in NC- and HFHS-fed WT mice**. A) Cladogram showing bacterial taxa significantly higher in the group of mice of the same colour, in the caecal microbiota. B) Indices of gut microbiota diversity, **P<0.01, ****P<0.0001, 2-way ANOVA followed by a 2-stage linear step-up procedure of Benjamini, Krieger and Yekutieli to correct for multiple comparisons by controlling the False Discovery Rate (<0.05). C) PCA of the gut microbiota, ***P<0.001 and table of pairwise comparisons (1-way PERMANOVA with Bonferroni correction). D) LDA score for predictive microbial pathway identified via PICRUSt [1], **P<0.01, with alpha value for the factorial Kruskal-Wallis test among classes and alpha value for the pairwise Wilcoxon test between subclasses set both at 0.01 and threshold on the logarithmic LDA score for discriminative features set at 2.0. E) PCA of the gut microbiome and table of pairwise comparisons (1-way PERMANOVA with Bonferroni correction). Data used to generate this PCA are those reported in Supppl.Fig.8D to generate LDA score. (“Lap” stands for laporotomized; “PF” stands for pair-feeding). “n” for: NC_WT_Lap = 4; NC_WT_GBP = 3; HFHS_WT_Lap = 9; HFHS_WT_Lap_PF = 10; HFHS_WT_GBP = 9.

**Supplementary Figure 9. Gastric bypass changes gut (caecum) microbiota and microbiome in NC- and HFHS-fed intestinal Glucose-6-Posphatase C KO mice**. A) Cladogram showing bacterial taxa significantly higher in the group of mice of the same colour, in the caecal microbiota. B) Indices of gut microbiota diversity, ****P<0.0001, 2-way ANOVA followed by a 2-stage linear step-up procedure of Benjamini, Krieger and Yekutieli to correct for multiple comparisons by controlling the False Discovery Rate (<0.05). C) PCA of the gut microbiota and table of pairwise comparisons (1-way PERMANOVA with Bonferroni correction). D) LDA score for predictive microbial pathway identified via PICRUSt [1], **P<0.01, with alpha value for the factorial Kruskal-Wallis test among classes and alpha value for the pairwise Wilcoxon test between subclasses set both at 0.01 and threshold on the logarithmic LDA score for discriminative features set at 3. E) PCA of the gut microbiome and table of pairwise comparisons (1-way PERMANOVA with Bonferroni correction). Data used to generate this PCA are those reported in Suppl.Fig.10D to generate LDA score. (“Lap” stands for laporotomized; “PF” stands for pair-feeding). “n” for: NC_iG6PC_KO_Lap = 5; NC_iG6PC_KO_GBP = 4; HFHS_iG6PC_KO_Lap = 6; HFHS_iG6PC_KO_Lap_PF = 6; HFHS_iG6PC_KO_GBP = 5.

**Supplementary Figure 10. Microbial taxa from gut (caecum) microbiota following gastric bypass in NC- and HFHS-fed WT and intestinal Glucose-6-Phosphatase C KO mice**. LDA score showing bacterial taxa significantly higher in the group of mice of the same colour, in the caecal microbiota of all groups of mice of the study (*the lack of group HFHS_iG6PC_KO_GBP means that this group has no higher bacterial taxon compared to any of the other groups of mice*). *P<0.05. “n” for: NC_WT_Lap = 4; NC_WT_GBP = 3; HFHS_WT_Lap = 9; HFHS_WT_Lap_PF = 10; HFHS_WT_GBP = 9; NC_iG6PC_KO_Lap = 5; NC_iG6PC_KO_GBP = 4; HFHS_iG6PC_KO_Lap = 6; HFHS_iG6PC_KO_Lap_PF = 6; HFHS_iG6PC_KO_GBP = 5.

**Supplementary Figure 11. Microbial functions from gut (caecum) microbiota following gastric bypass in NC- and HFHS-fed WT and intestinal Glucose-6-Posphatase C KO mice**. A-D) LDA score for predictive microbial pathway identified via PICRUSt [1], **P<0.01, with alpha value for the factorial Kruskal-Wallis test among classes and alpha value for the pairwise Wilcoxon test between subclasses set both at 0.01 and threshold on the logarithmic LDA score for discriminative features set at 2. “n” for: NC_WT_Lap = 4; NC_WT_GBP = 3; HFHS_WT_Lap = 9; HFHS_WT_Lap_PF = 10; HFHS_WT_GBP = 9; NC_iG6PC_KO_Lap = 5; NC_iG6PC_KO_GBP = 4; HFHS_iG6PC_KO_Lap = 6; HFHS_iG6PC_KO_Lap_PF = 6; HFHS_iG6PC_KO_GBP = 5.

**Supplementary figures legends references**

[1] Langille MG, Zaneveld J, Caporaso JG, McDonald D, Knights D, Reyes JA, Clemente JC, Burkepile DE, Vega Thurber RL, Knight R, Beiko RG, Huttenhower C. Predictive functional profiling of microbial communities using 16S rRNA marker gene sequences. Nat Biotechnol 2013;31(9):814-21.
