# Supplementary material for: Intestinal gluconeogenesis shapes gut microbiota, fecal and urine metabolome in mice with gastric bypass surgery
Source: Sci Rep. 2022 Jan 26;12:1415. doi: 10.1038/s41598-022-04902-y (PMC8791999; doi:10.1038/s41598-022-04902-y)
Supplement: Supplementary file 2 — Supplementary Figures. [file 41598_2022_4902_MOESM2_ESM.pdf]

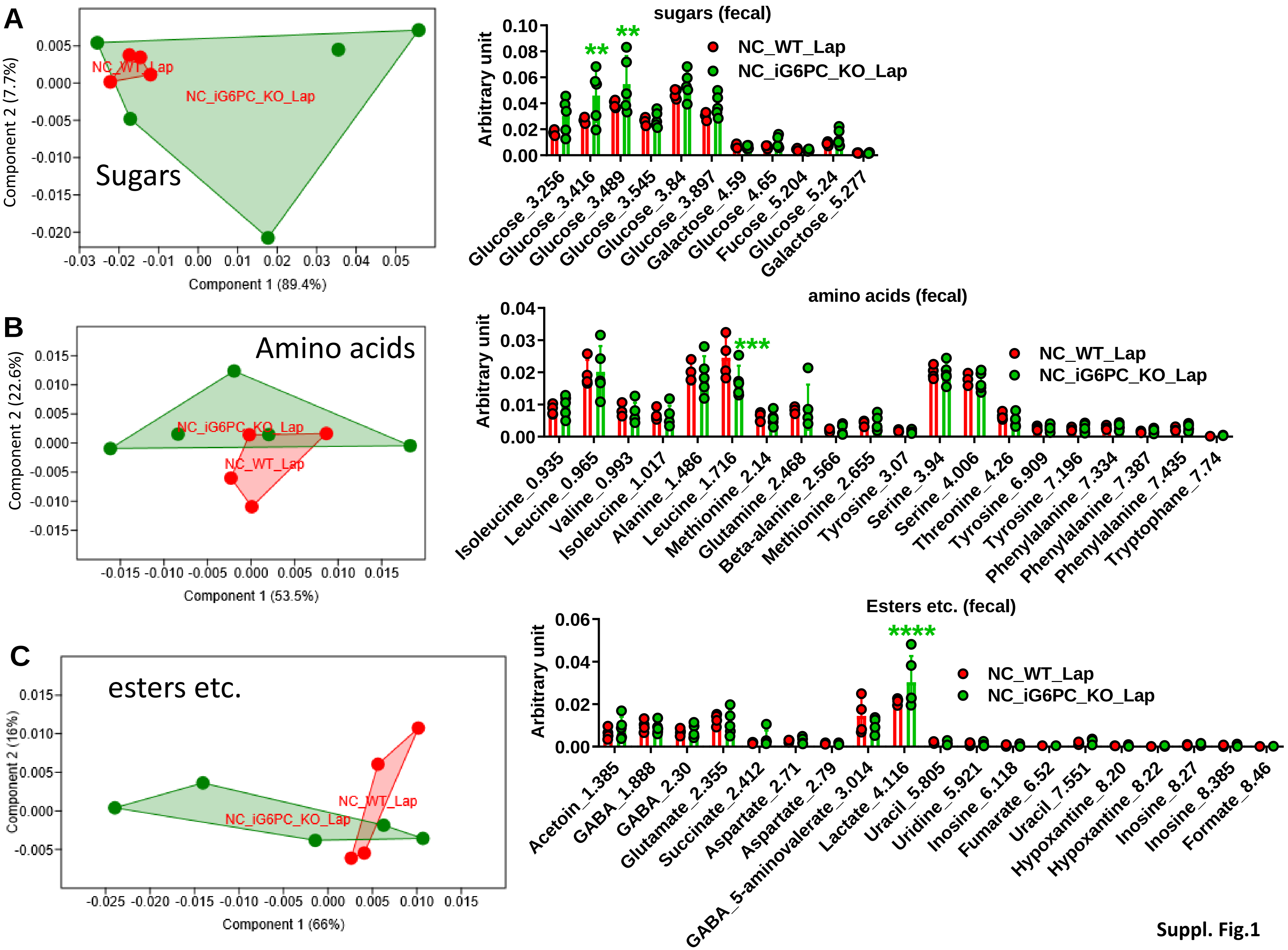
A
Sugars
NC_WT_Lap
NC_iG6PC_KO_Lap
Component 2 (7.7%)
Component 1 (89.4%)
sugars (fecal)
Arbitrary unit
NC_WT_Lap
NC_iG6PC_KO_Lap
Glucose_3.256
Glucose_3.416
Glucose_3.489
Glucose_3.545
Glucose_3.84
Glucose_3.897
Galactose_4.59
Glucose_4.65
Fucose_5.204
Glucose_5.24
Galactose_5.277
B
Amino acids
NC_iG6PC_KO_Lap
NC_WT_Lap
Component 2 (22.6%)
Component 1 (53.5%)
amino acids (fecal)
Arbitrary unit
NC_WT_Lap
NC_iG6PC_KO_Lap
Isoleucine_0.935
Leucine_0.965
Valine_0.993
Isoleucine_1.017
Alanine_1.486
Leucine_1.716
Methionine_2.14
Glutamine_2.468
Beta-alanine_2.566
Methionine_2.655
Tyrosine_3.07
Serine_3.94
Serine_4.006
Threonine_4.26
Tyrosine_6.909
Tyrosine_7.196
Phenylalanine_7.334
Phenylalanine_7.387
Phenylalanine_7.435
Tryptophane_7.74
C
esters etc.
NC_WT_Lap
NC_iG6PC_KO_Lap
Component 2 (16%)
Component 1 (66%)
Esters etc. (fecal)
Arbitrary unit
NC_WT_Lap
NC_iG6PC_KO_Lap
Acetoin_1.385
GABA_1.888
GABA_2.30
Glutamate_2.355
Succinate_2.412
Aspartate_2.71
Aspartate_2.79
GABA_5-aminovalerate_3.014
Lactate_4.116
Uracil_5.805
Uridine_5.921
Inosine_6.118
Fumarate_6.52
Uracil_7.551
Hypoxantine_8.20
Hypoxantine_8.22
Inosine_8.27
Inosine_8.385
Formate_8.46

Suppl. Fig.1

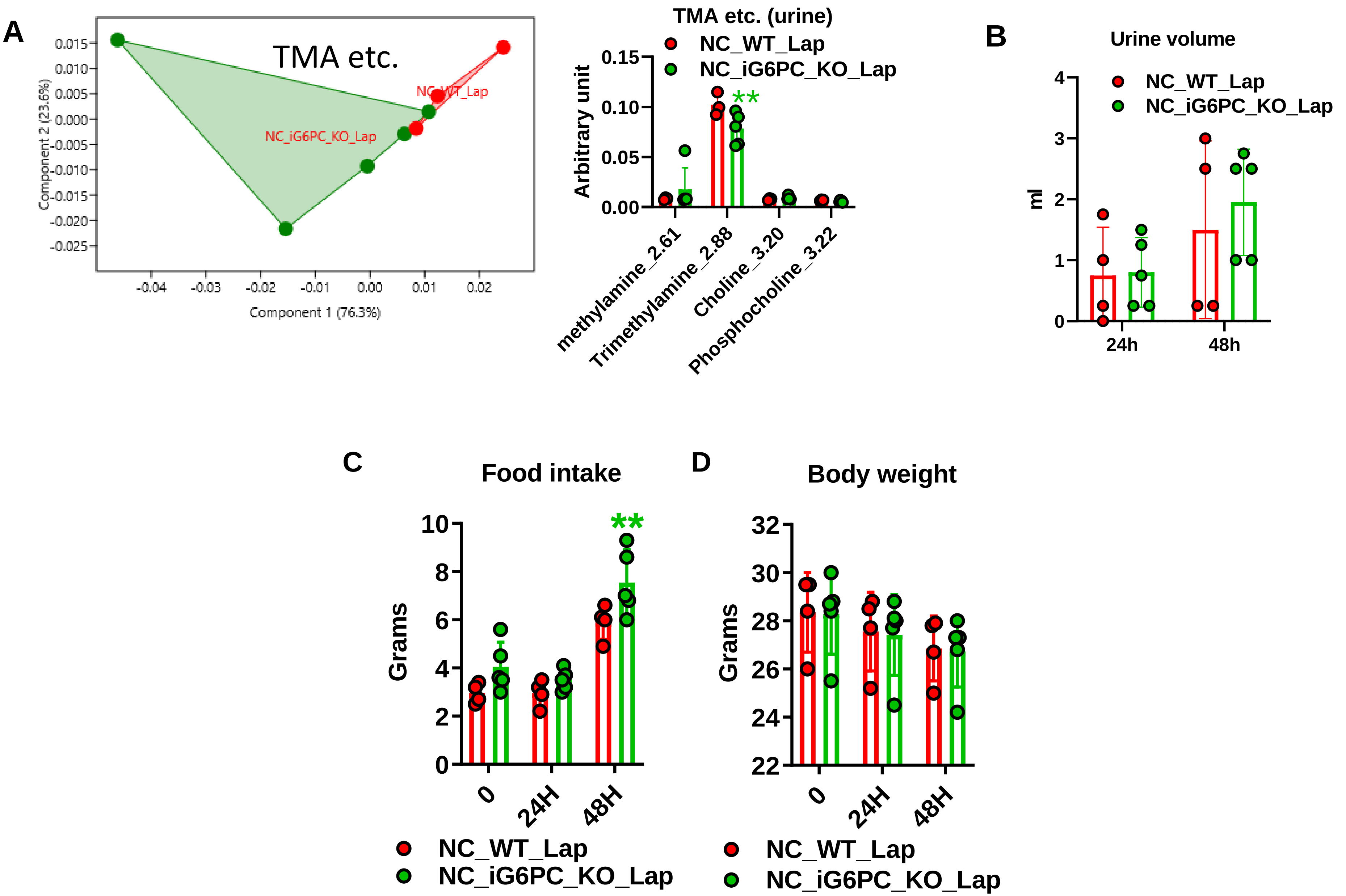
A
TMA etc.
NC_WT_Lap
NC_iG6PC_KO_Lap
Component 1 (76.3%)
Component 2 (23.6%)
TMA etc. (urine)
NC_WT_Lap
NC_iG6PC_KO_Lap
Arbitrary unit
**
methylamine_2.61
Trimethylamine_2.88
Choline_3.20
Phosphocholine_3.22
B
Urine volume
NC_WT_Lap
NC_iG6PC_KO_Lap
ml
24h
48h
C
Food intake
Grams
**
0
24H
48H
NC_WT_Lap
NC_iG6PC_KO_Lap
D
Body weight
Grams
0
24H
48H
NC_WT_Lap
NC_iG6PC_KO_Lap

Suppl. Fig.2

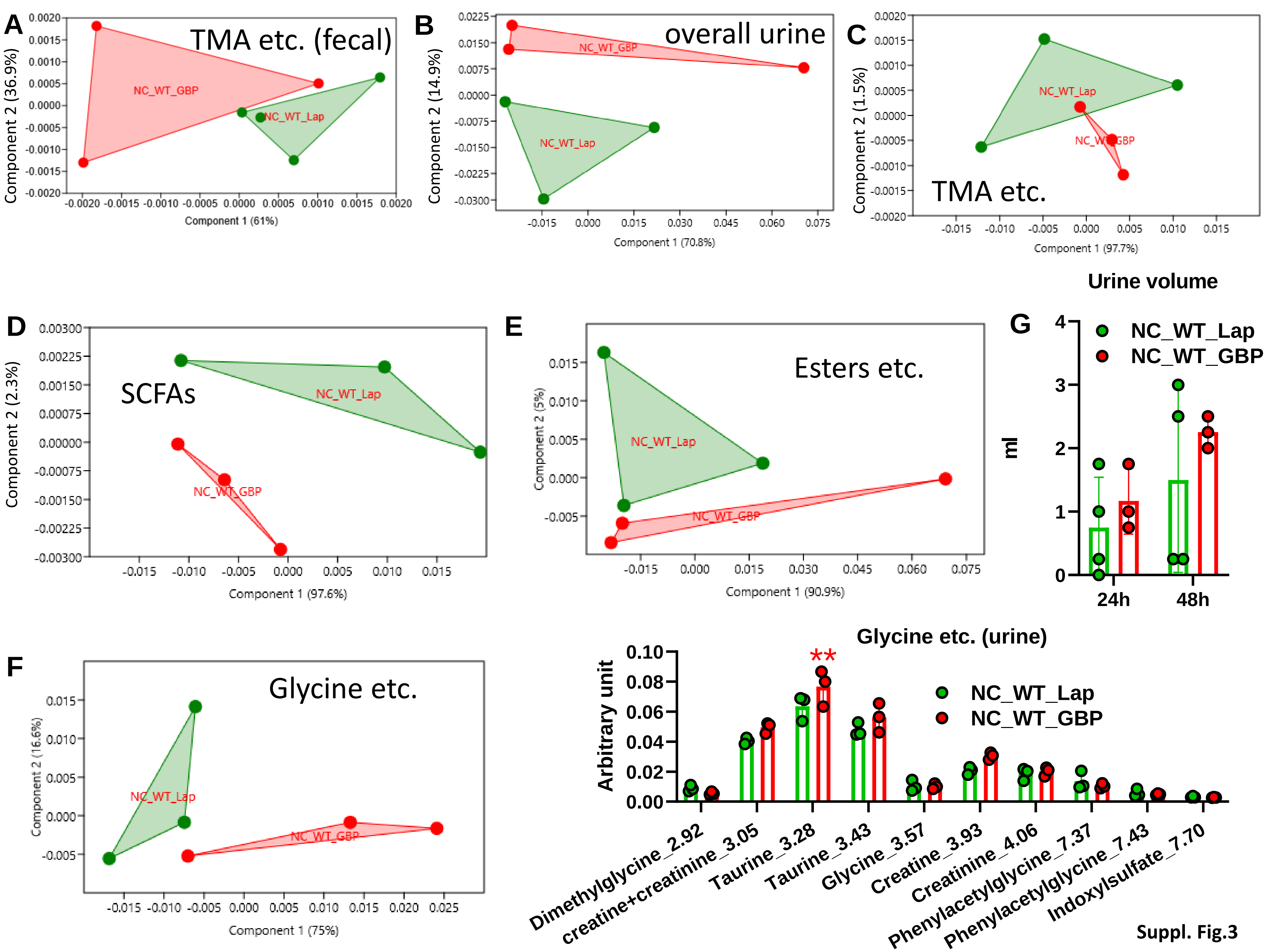
A
TMA etc. (fecal)
NC_WT_GBP
NC_WT_Lap
Component 2 (36.9%)
Component 1 (61%)
B
overall urine
NC_WT_GBP
NC_WT_Lap
Component 2 (14.9%)
Component 1 (70.8%)
C
TMA etc.
NC_WT_Lap
NC_WT_GBP
Component 2 (1.5%)
Component 1 (97.7%)
D
SCFAs
NC_WT_Lap
NC_WT_GBP
Component 2 (2.3%)
Component 1 (97.6%)
E
Esters etc.
NC_WT_Lap
NC_WT_GBP
Component 2 (5%)
Component 1 (90.9%)
G
Urine volume
NC_WT_Lap
NC_WT_GBP
ml
24h
48h
F
Glycine etc.
NC_WT_Lap
NC_WT_GBP
Component 2 (16.6%)
Component 1 (75%)
Glycine etc. (urine)
Arbitrary unit
**
NC_WT_Lap
NC_WT_GBP
Dimethylglycine_2.92
creatine+creatinine_3.05
Taurine_3.28
Taurine_3.43
Glycine_3.57
Creatine_3.93
Creatinine_4.06
Phenylacetylglycine_7.37
Phenylacetylglycine_7.43
Indoxylsulfate_7.70

Suppl. Fig.3

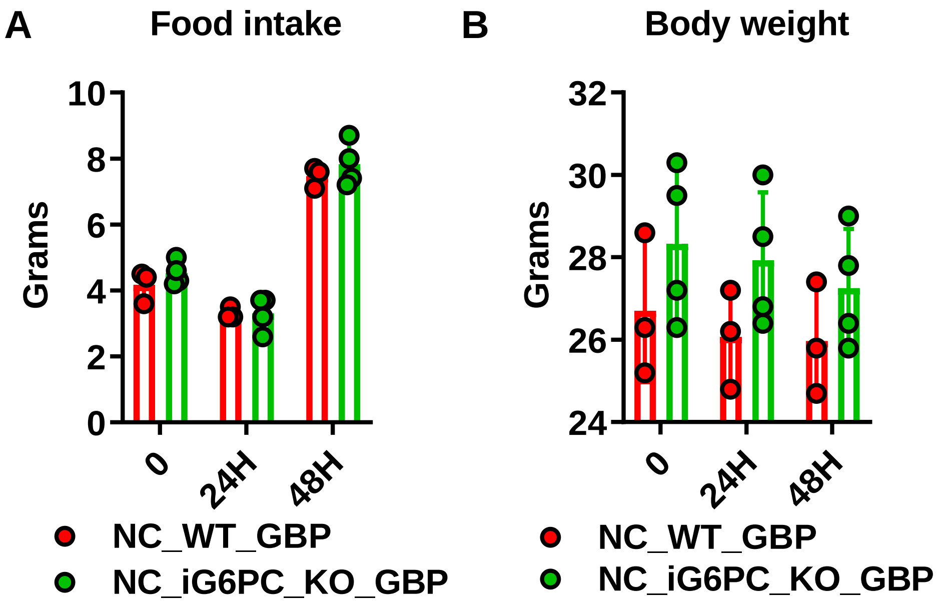
A
Food intake
10
8
6
4
2
0
Grams
0
24H
48H
NC_WT_GBP
NC_iG6PC_KO_GBP
B
Body weight
32
30
28
26
24
Grams
0
24H
48H
NC_WT_GBP
NC_iG6PC_KO_GBP

Suppl. Fig.4

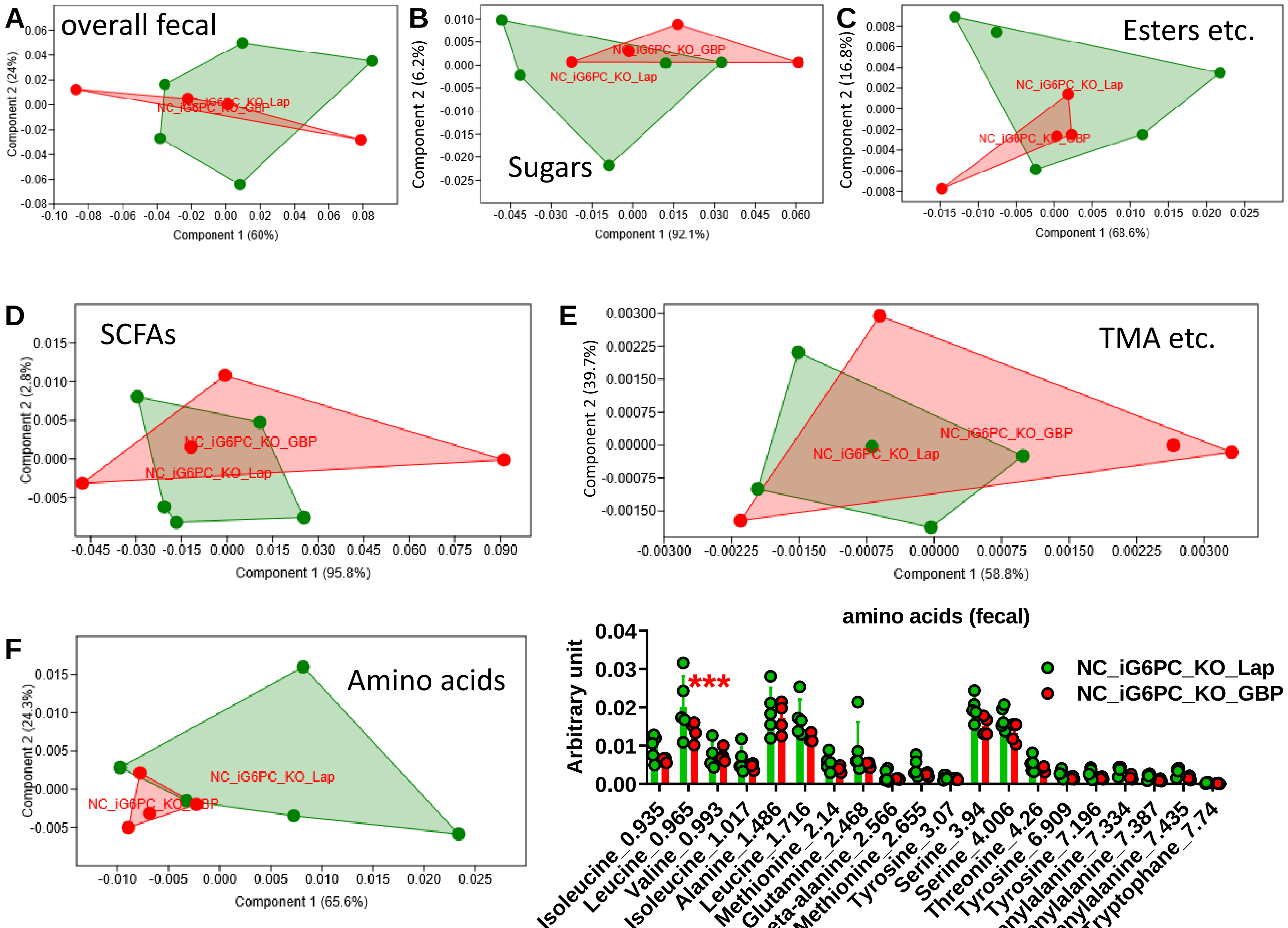

A
overall fecal
Component 2 (24%)
Component 1 (60%)
NC_iG6PC_KO_Lap
NC_iG6PC_KO_GBP
B
Sugars
Component 2 (6.2%)
Component 1 (92.1%)
NC_iG6PC_KO_GBP
NC_iG6PC_KO_Lap
C
Esters etc.
Component 2 (16.8%)
Component 1 (68.6%)
NC_iG6PC_KO_Lap
NC_iG6PC_KO_GBP
D
SCFAs
Component 2 (2.8%)
Component 1 (95.8%)
NC_iG6PC_KO_GBP
NC_iG6PC_KO_Lap
E
TMA etc.
Component 2 (39.7%)
Component 1 (58.8%)
NC_iG6PC_KO_GBP
NC_iG6PC_KO_Lap
F
Amino acids
Component 2 (24.3%)
Component 1 (65.6%)
NC_iG6PC_KO_Lap
NC_iG6PC_KO_GBP
amino acids (fecal)
Arbitrary unit
***
NC_iG6PC_KO_Lap
NC_iG6PC_KO_GBP
Isoleucine_0.935
Leucine_0.965
Valine_0.993
Isoleucine_1.017
Alanine_1.486
Leucine_1.716
Methionine_2.14
Glutamine_2.468
Beta-alanine_2.566
Methionine_2.655
Tyrosine_3.07
Serine_3.94
Threonine_4.006
Tyrosine_4.26
Tyrosine_6.909
Phenylalanine_7.196
Phenylalanine_7.334
Phenylalanine_7.387
Tryptophane_7.435
Tryptophane_7.74

Suppl. Fig.5

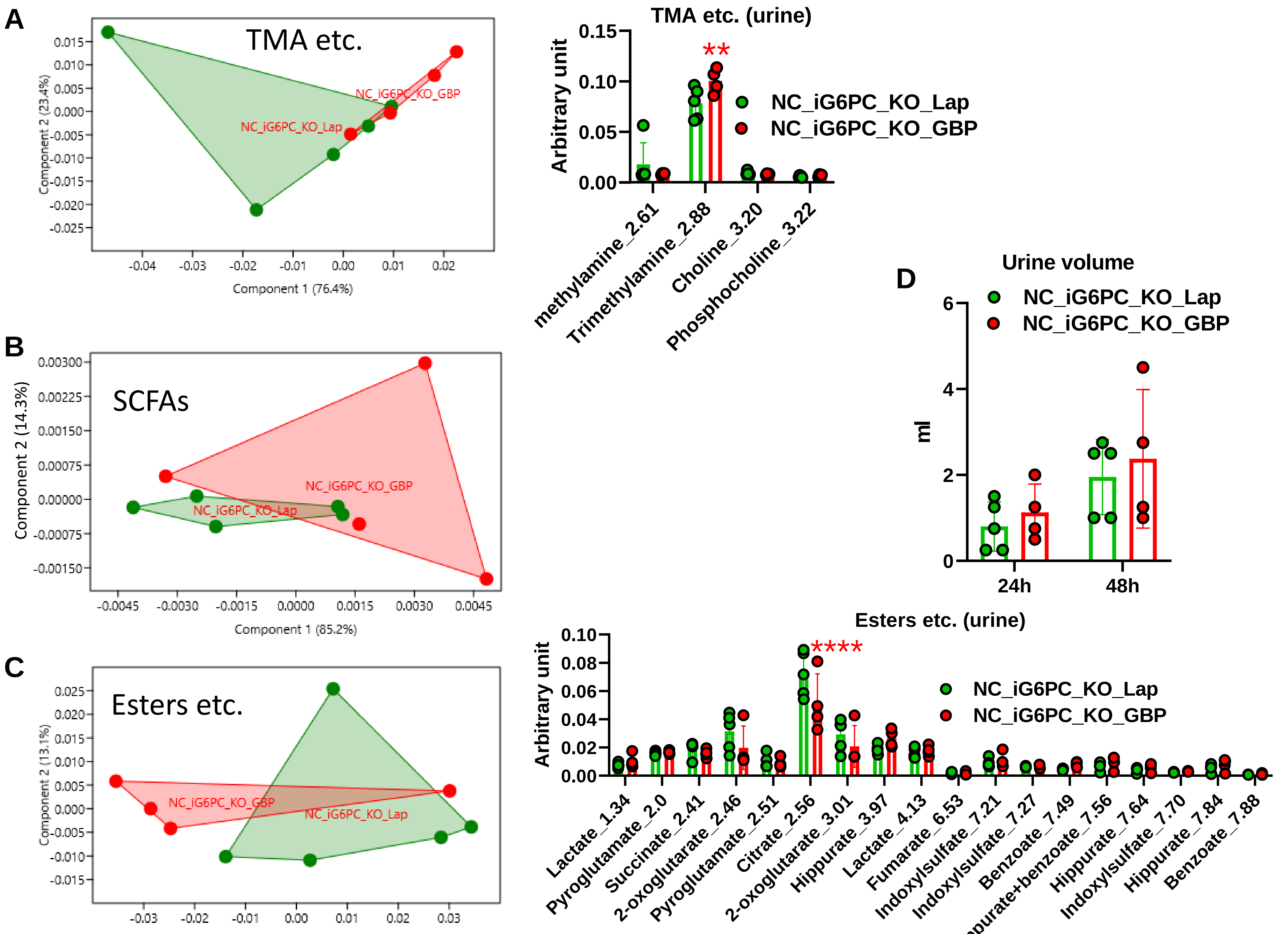
A
TMA etc.
NC_iG6PC_KO_GBP
NC_iG6PC_KO_Lap
Component 2 (23.4%)
Component 1 (76.4%)
TMA etc. (urine)
Arbitrary unit
**
NC_iG6PC_KO_Lap
NC_iG6PC_KO_GBP
methylamine_2.61
Trimethylamine_2.88
Choline_3.20
Phosphocholine_3.22
B
SCFAs
NC_iG6PC_KO_GBP
NC_iG6PC_KO_Lap
Component 2 (14.3%)
Component 1 (85.2%)
D
Urine volume
NC_iG6PC_KO_Lap
NC_iG6PC_KO_GBP
ml
24h
48h
C
Esters etc.
NC_iG6PC_KO_GBP
NC_iG6PC_KO_Lap
Component 2 (13.1%)
Component 1 (77.2%)
Esters etc. (urine)
Arbitrary unit
****
NC_iG6PC_KO_Lap
NC_iG6PC_KO_GBP
Lactate_1.34
Pyroglutamate_2.0
Succinate_2.41
2-oxoglutarate_2.46
Pyroglutamate_2.51
Citrate_2.56
2-oxoglutarate_3.01
Hippurate_3.97
Lactate_4.13
Fumarate_6.53
Indoxylsulfate_7.21
Indoxylsulfate_7.27
Benzoate_7.49
Hippurate+benzoate_7.56
Hippurate_7.64
Indoxylsulfate_7.70
Hippurate_7.84
Benzoate_7.88

Suppl. Fig.6

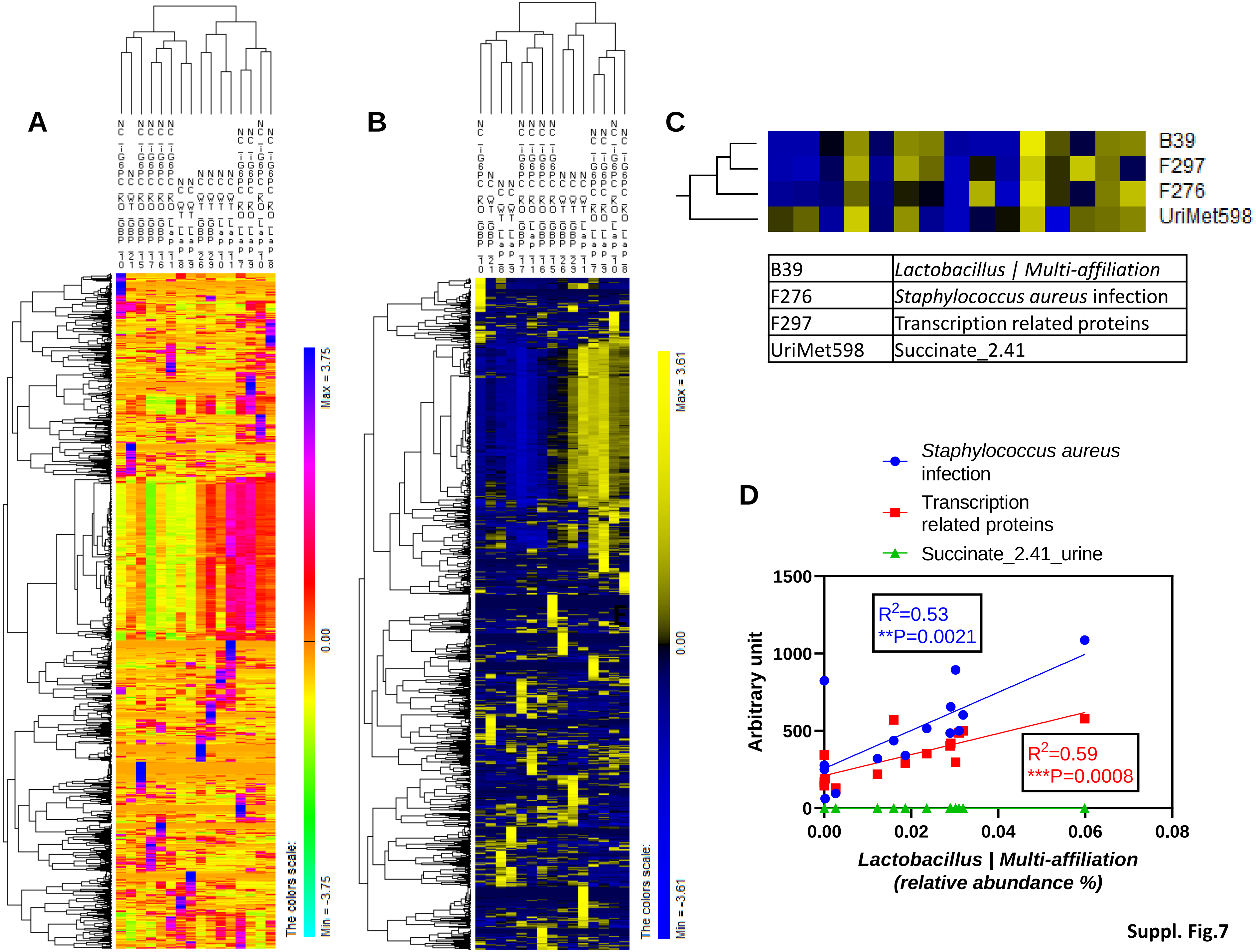

A
B
C
D
The colors scale:
Max = 3.75
0.00
Min = -3.75
The colors scale:
Max = 3.61
0.00
Min = -3.61
B39
F297
F276
UriMet598
B39 | Lactobacillus | Multi-affiliation
F276 | Staphylococcus aureus infection
F297 | Transcription related proteins
UriMet598 | Succinate_2.41
Staphylococcus aureus infection
Transcription related proteins
Succinate_2.41_urine
R2=0.53
**P=0.0021
R2=0.59
***P=0.0008
Arbitrary unit
1500
1000
500
0
0.00
0.02
0.04
0.06
0.08
Lactobacillus | Multi-affiliation
(relative abundance %)

Suppl. Fig.7

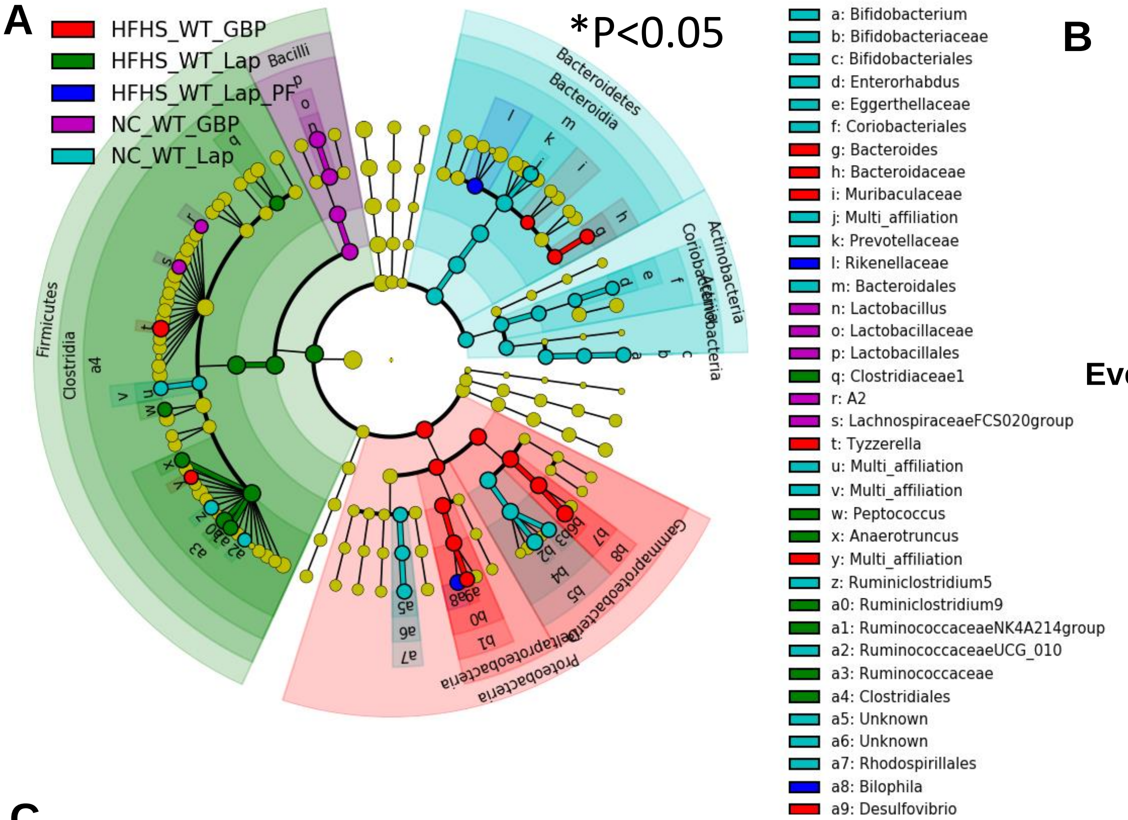

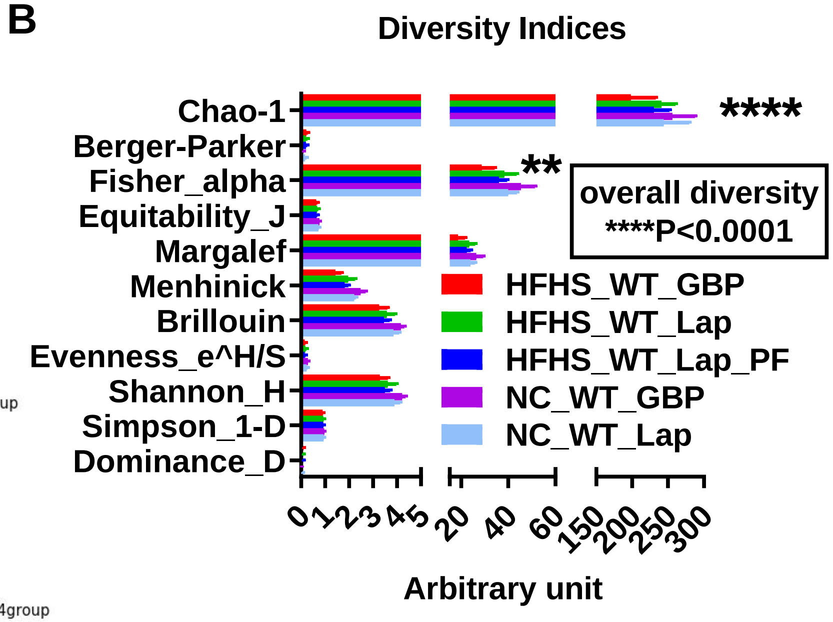

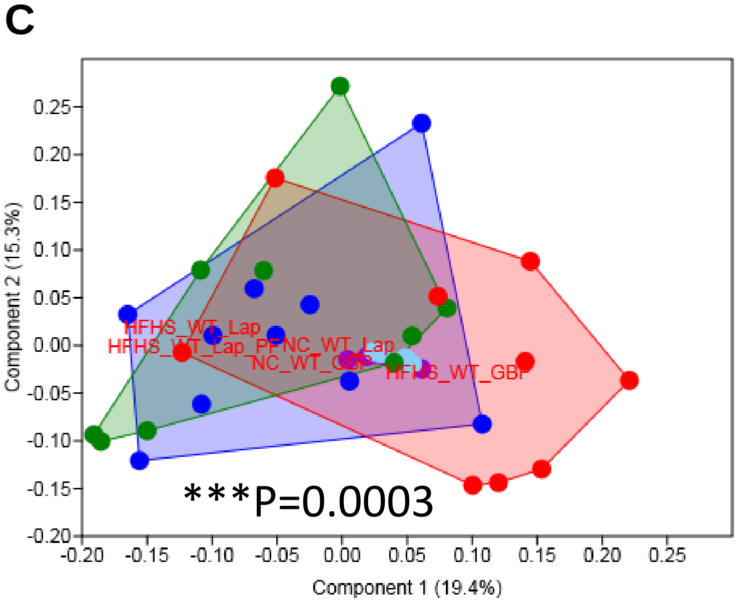

	HFHS_WT_Lap_PF	HFHS_WT_GBP	HFHS_WT_Lap	NC_WT_Lap	NC_WT_GBP
HFHS_WT_Lap_PF		**0.006**	1	***0.011**	***0.042**
HFHS_WT_GBP	****0.006**		****0.002**	***0.015**	***0.049**
HFHS_WT_Lap	1	****0.002**		***0.024**	***0.046**
NC_WT_Lap	***0.011**	***0.015**	***0.024**		0.816
NC_WT_GBP	***0.042**	***0.049**	***0.046**	0.816	

Suppl. Fig.8

D

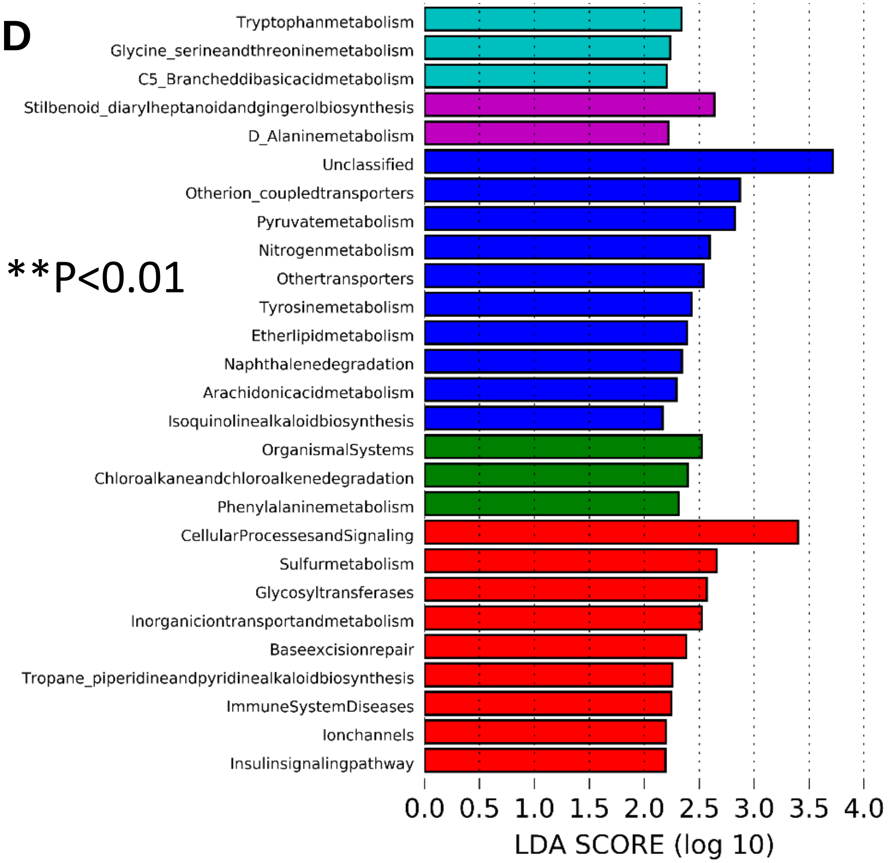

**P<0.01

E

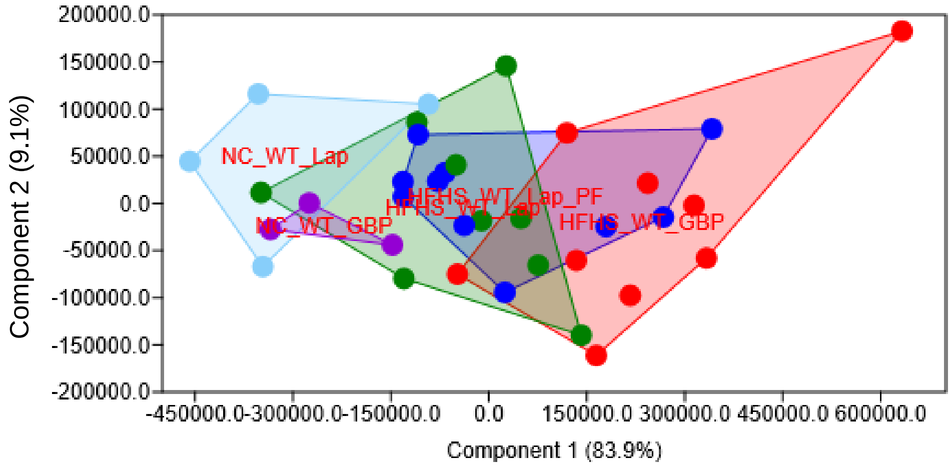

	HFHS_WT_Lap_PF	HFHS_WT_GBP	HFHS_WT_Lap	NC_WT_Lap	NC_WT_GBP
HFHS_WT_Lap_PF		0.211	1	0.057	0.067
HFHS_WT_GBP	0.211		***0.018**	***0.033**	***0.042**
HFHS_WT_Lap	1	***0.018**		0.155	0.207
NC_WT_Lap	0.057	***0.033**	0.155		1
NC_WT_GBP	0.067	***0.042**	0.207	1	

Suppl. Fig.8

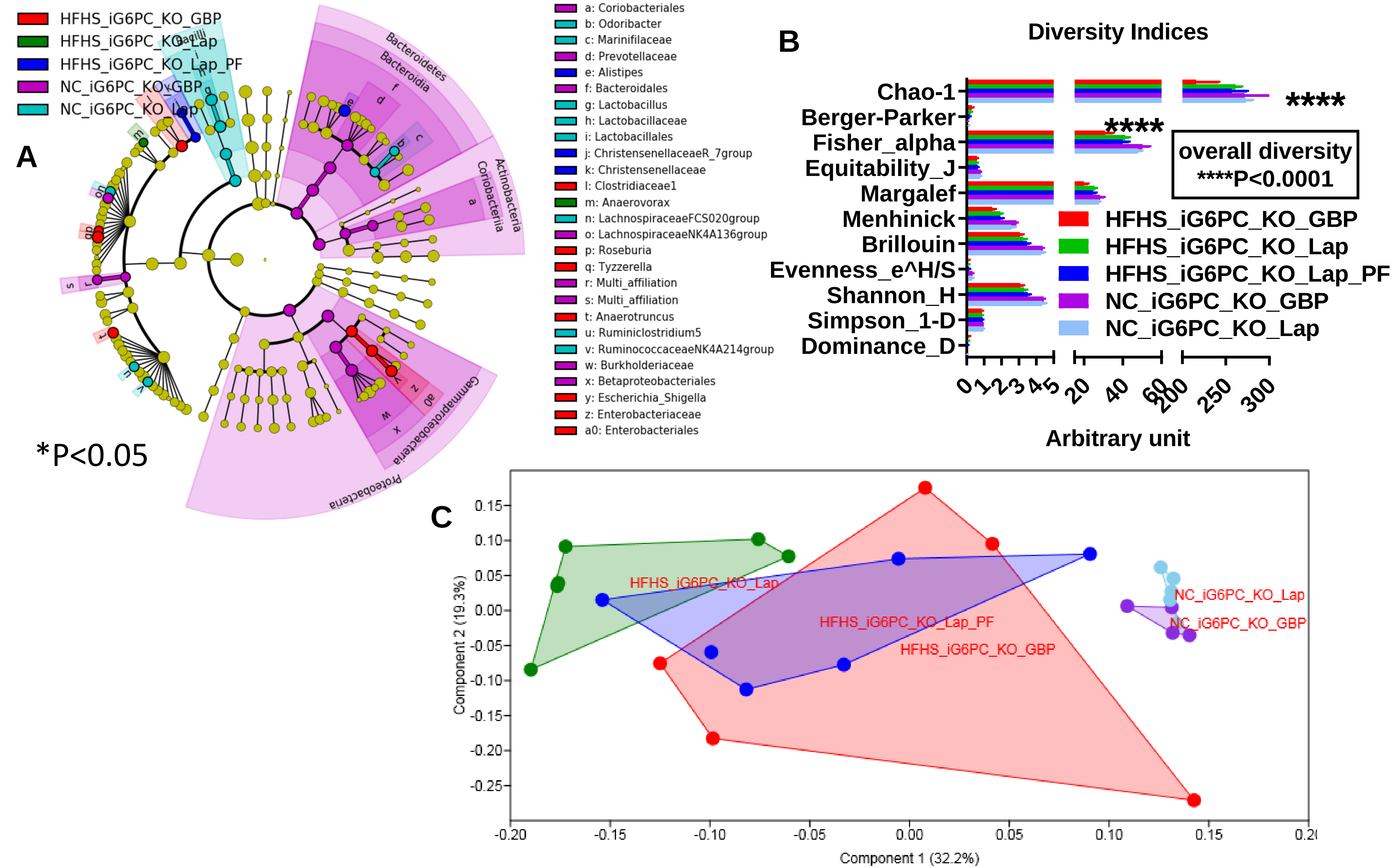

	HFHS_iG6PC_KO_GBP	NC_iG6PC_KO_GBP	NC_iG6PC_KO_Lap	HFHS_iG6PC_KO_Lap	HFHS_iG6PC_KO_Lap_PF
HFHS_iG6PC_KO_GBP		0.064	0.085	***0.016**	0.087
NC_iG6PC_KO_GBP	0.064		0.082	***0.046**	***0.049**
NC_iG6PC_KO_Lap	0.085	0.082		***0.031**	***0.034**
HFHS_iG6PC_KO_Lap	***0.016**	***0.046**	***0.031**		0.676
HFHS_iG6PC_KO_Lap_PF		0.087	***0.049**	***0.034**	0.676

Suppl. Fig.9

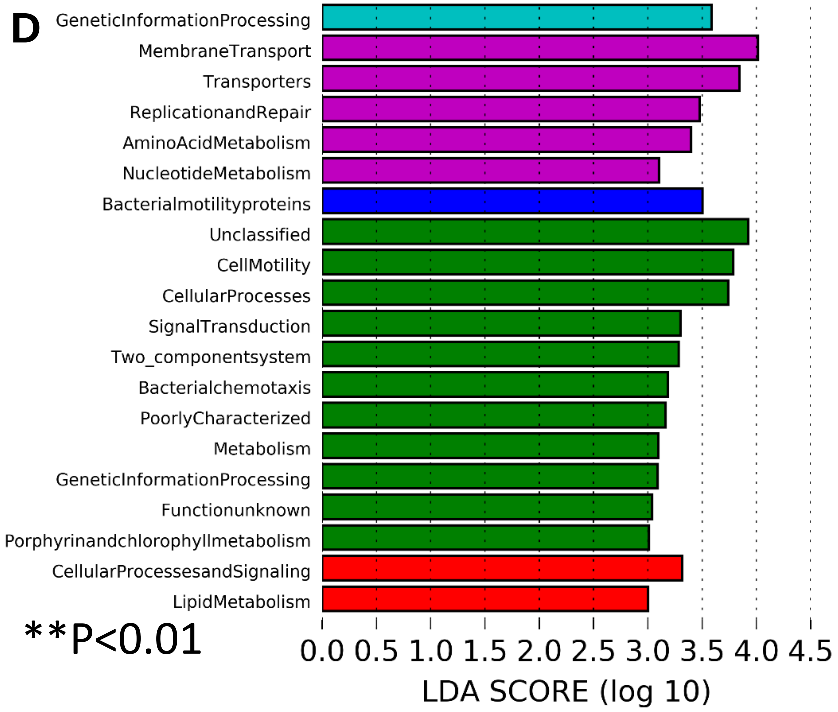

**P<0.01

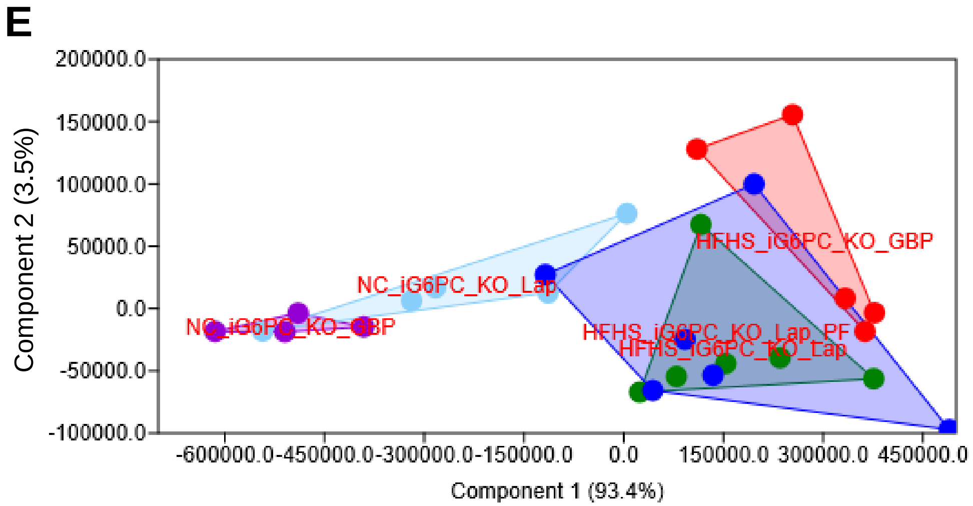

	HFHS_iG6PC_KO_GBP	NC_iG6PC_KO_GBP	NC_iG6PC_KO_Lap	HFHS_iG6PC_KO_Lap	HFHS_iG6PC_KO_Lap_PF
HFHS_iG6PC_KO_GBP		0.065	0.06	0.48	0.919
NC_iG6PC_KO_GBP	0.065		0.559	***0.042**	***0.044**
NC_iG6PC_KO_Lap	0.06	0.559		***0.026**	0.114
HFHS_iG6PC_KO_Lap	0.48	***0.042**	***0.026**		1
HFHS_iG6PC_KO_Lap_PF		0.919	***0.044**	0.114	1

Suppl. Fig.9

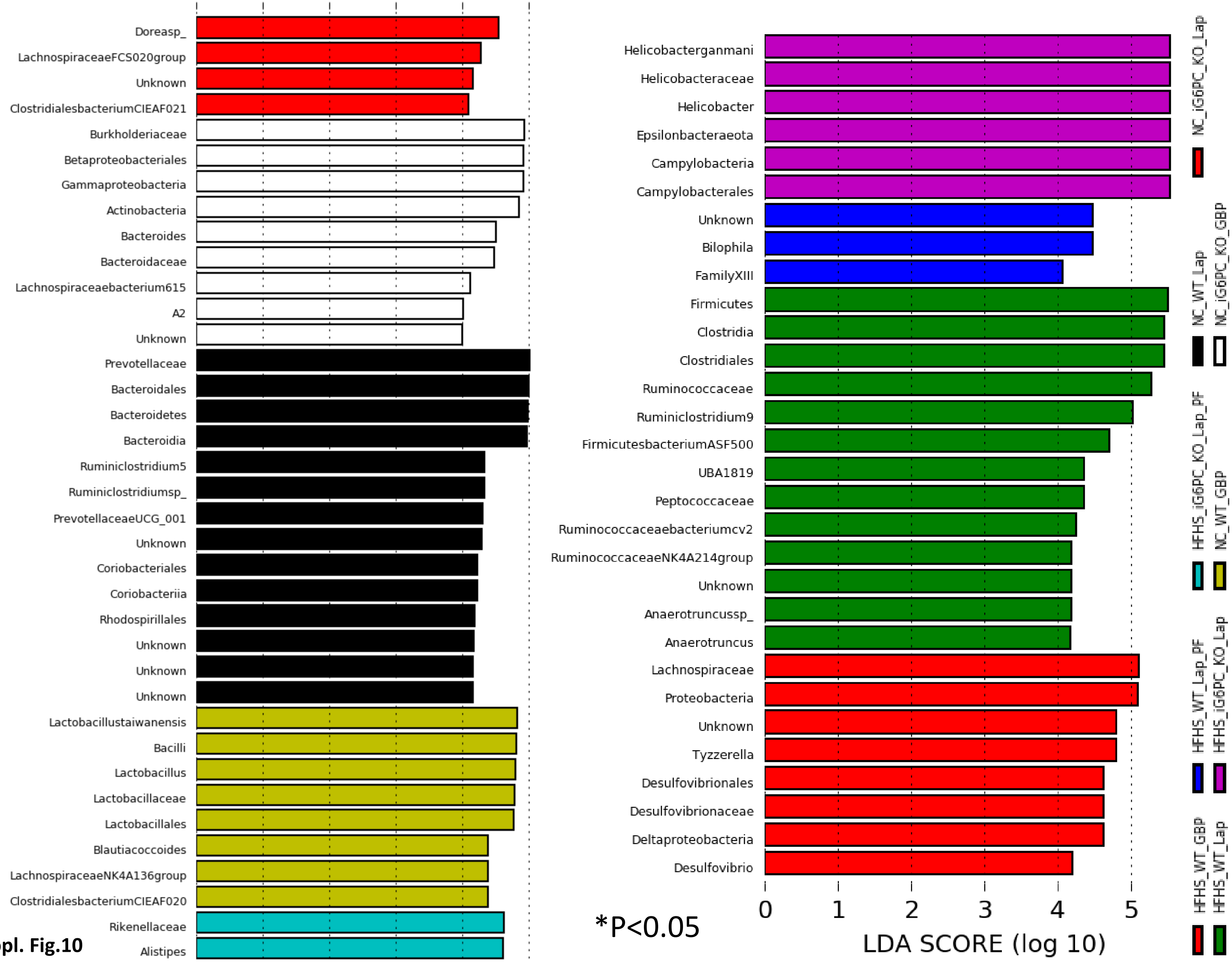
Doreasp_
LachnospiraceaeFCS020group
Unknown
ClostridialesbacteriumCIEAF021
Burkholderiaceae
Betaproteobacteriales
Gammaproteobacteria
Actinobacteria
Bacteroides
Bacteroidaceae
Lachnospiraceaebacterium615
A2
Unknown
Prevotellaceae
Bacteroidales
Bacteroidetes
Bacteroidia
Ruminiclostridium5
Ruminiclostridiumsp_
PrevotellaceaeUCG_001
Unknown
Coriobacteriales
Coriobacteriia
Rhodospirillales
Unknown
Unknown
Unknown
Lactobacillustaiwanensis
Bacilli
Lactobacillus
Lactobacillaceae
Lactobacillales
Blautiacoccoides
LachnospiraceaeNK4A136group
ClostridialesbacteriumCIEAF020
Rikenellaceae
Alistipes
Helicobacterganmani
Helicobacteraceae
Helicobacter
Epsilonbacteraeota
Campylobacteria
Campylobacterales
Unknown
Bilophila
FamilyXIII
Firmicutes
Clostridia
Clostridiales
Ruminococcaceae
Ruminiclostridium9
FirmicutesbacteriumASF500
UBA1819
Peptococcaceae
Ruminococcaceaebacteriumcv2
RuminococcaceaeNK4A214group
Unknown
Anaerotruncussp_
Anaerotruncus
Lachnospiraceae
Proteobacteria
Unknown
Tyzzerella
Desulfovibrionales
Desulfovibrionaceae
Deltaproteobacteria
Desulfovibrio
0
1
2
3
4
5
LDA SCORE (log 10)
*P<0.05
HFHS_WT_GBP
HFHS_WT_Lap
HFHS_WT_Lap_PF
HFHS_iG6PC_KO_Lap
HFHS_iG6PC_KO_Lap_PF
NC_WT_GBP
NC_WT_Lap
NC_iG6PC_KO_GBP
NC_iG6PC_KO_Lap

Suppl. Fig.10

A

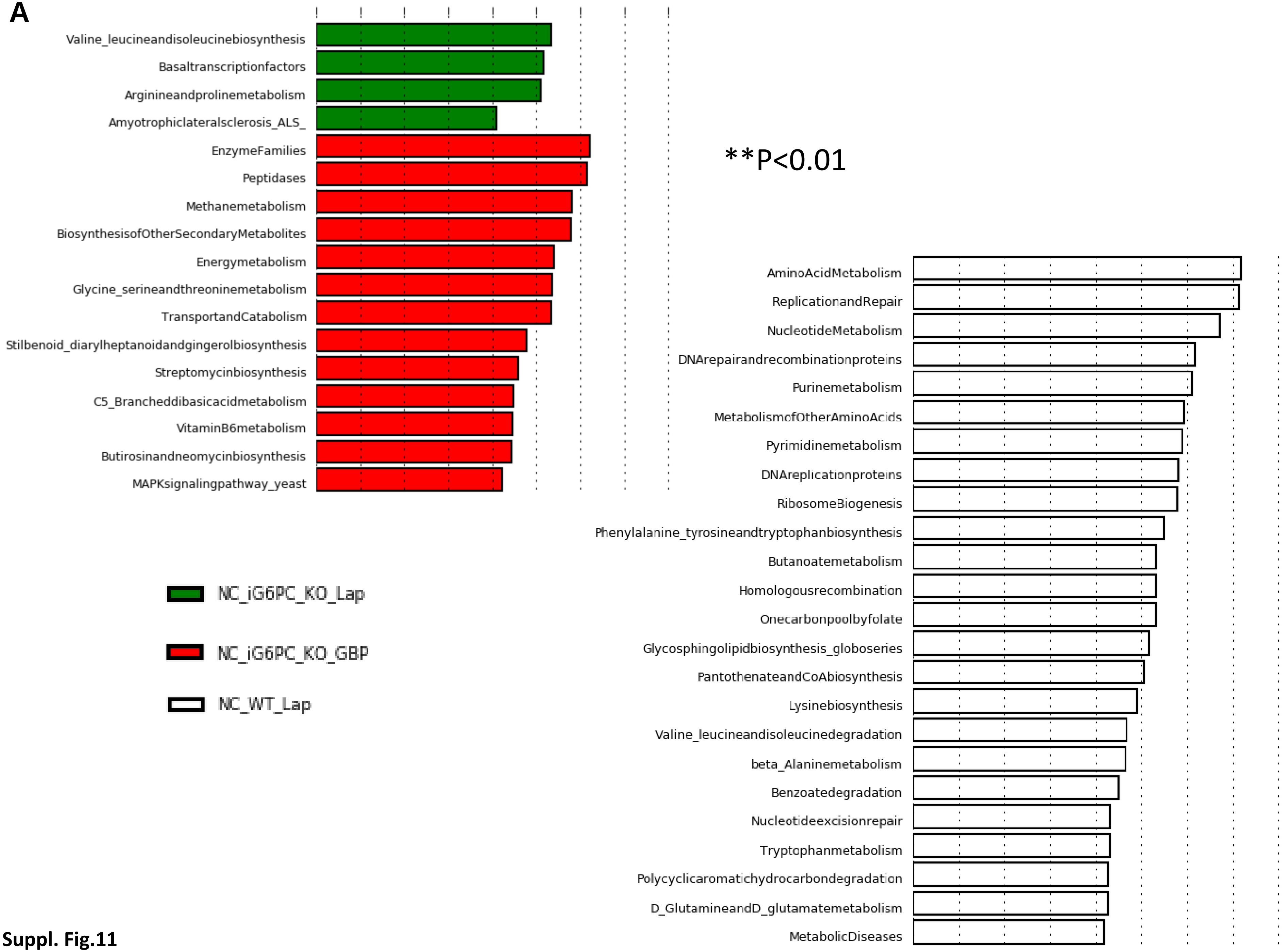

Suppl. Fig.11

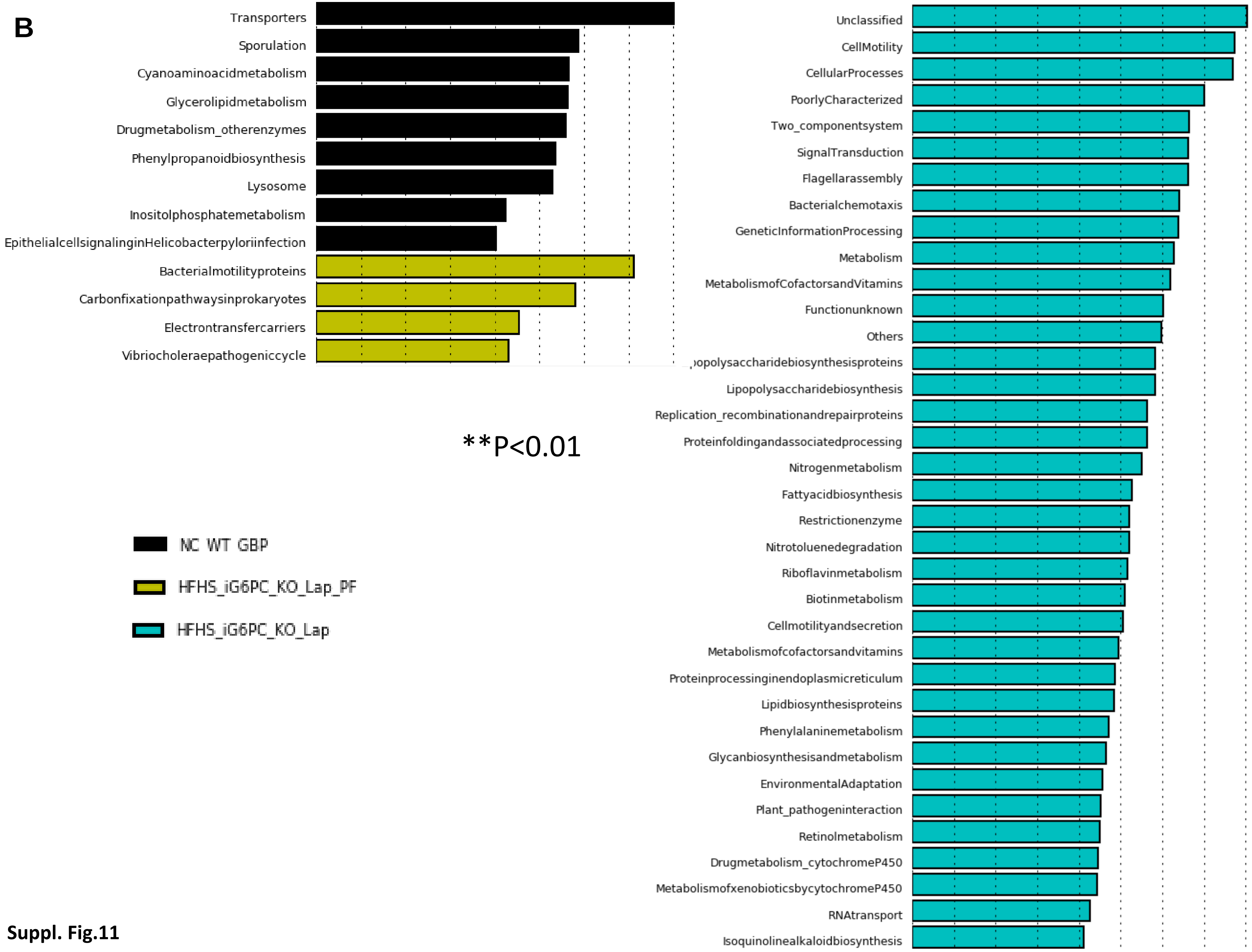

B
Transporters
Sporulation
Cyanoaminoacidmetabolism
Glycerolipidmetabolism
Drugmetabolism_otherenzymes
Phenylpropanoidbiosynthesis
Lysosome
Inositolphosphatemetabolism
EpithelialcellsignalinginHelicobacterpyloriinfection
Bacterialmotilityproteins
Carbonfixationpathwaysinprokaryotes
Electrontransfercarriers
Vibriocholeraepathogeniccycle
**P<0.01
NC_WT_GBP
HFHS_iG6PC_KO_Lap_PF
HFHS_iG6PC_KO_Lap
Unclassified
CellMotility
CellularProcesses
PoorlyCharacterized
Two_componentsystem
SignalTransduction
Flagellarassembly
Bacterialchemotaxis
GeneticInformationProcessing
Metabolism
MetabolismofCofactorsandVitamins
Functionunknown
Others
popolysaccharidebiosynthesisproteins
Lipopolysaccharidebiosynthesis
Replication_recombinationandrepairproteins
Proteinfoldingandassociatedprocessing
Nitrogenmetabolism
Fattyacidbiosynthesis
Restrictionenzyme
Nitrotoluenedegradation
Riboflavinmetabolism
Biotinmetabolism
Cellmotilityandsecretion
Metabolismofcofactorsandvitamins
Proteinprocessinginendoplasmicreticulum
Lipidbiosynthesisproteins
Phenylalaninemetabolism
Glycanbiosynthesisandmetabolism
EnvironmentalAdaptation
Plant_pathogeninteraction
Retinolmetabolism
Drugmetabolism_cytochromeP450
MetabolismofxenobioticsbycytochromeP450
RNAtransport
Isoquinolinealkaloidbiosynthesis

Suppl. Fig.11

C

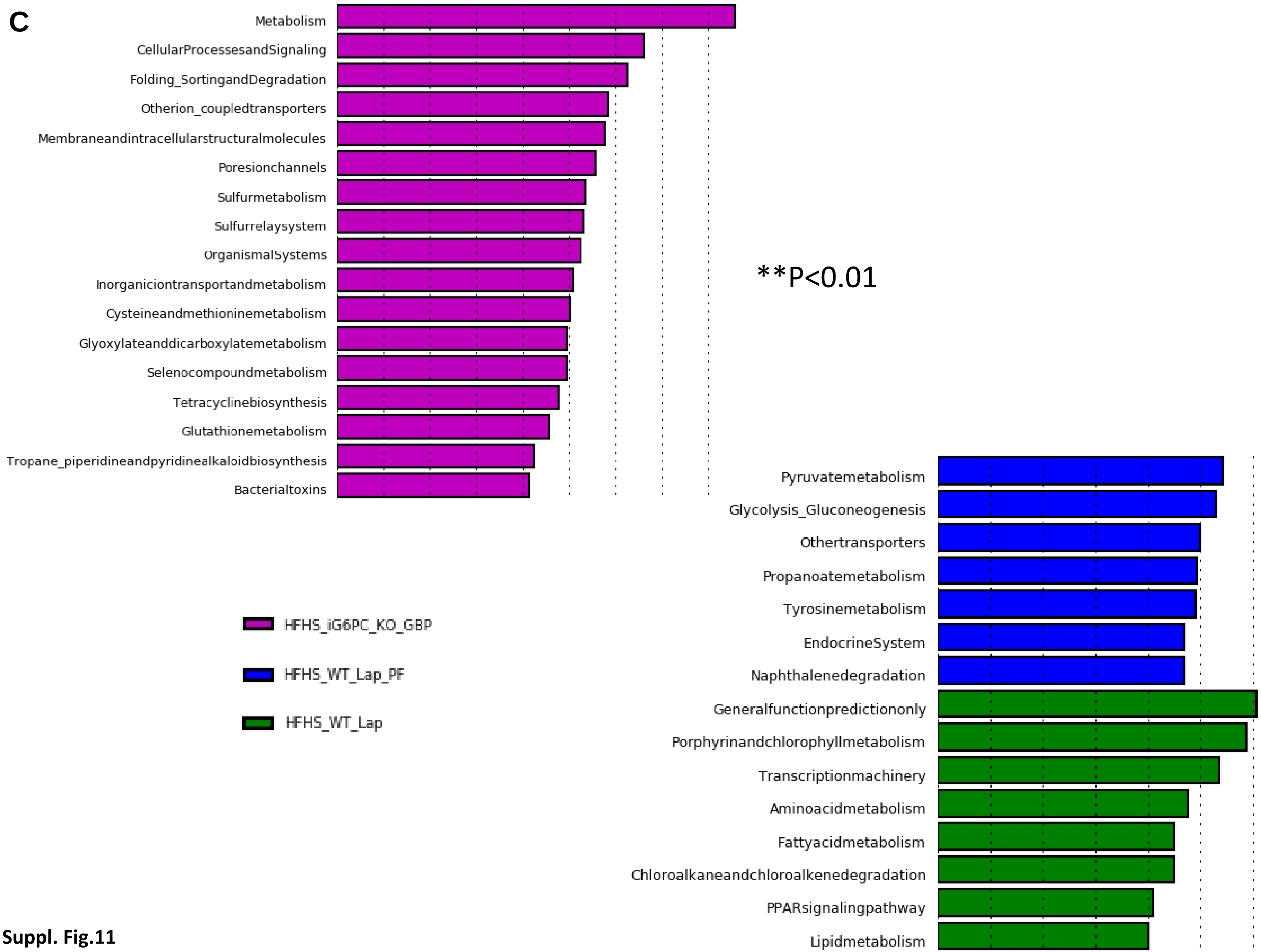

Suppl. Fig.11

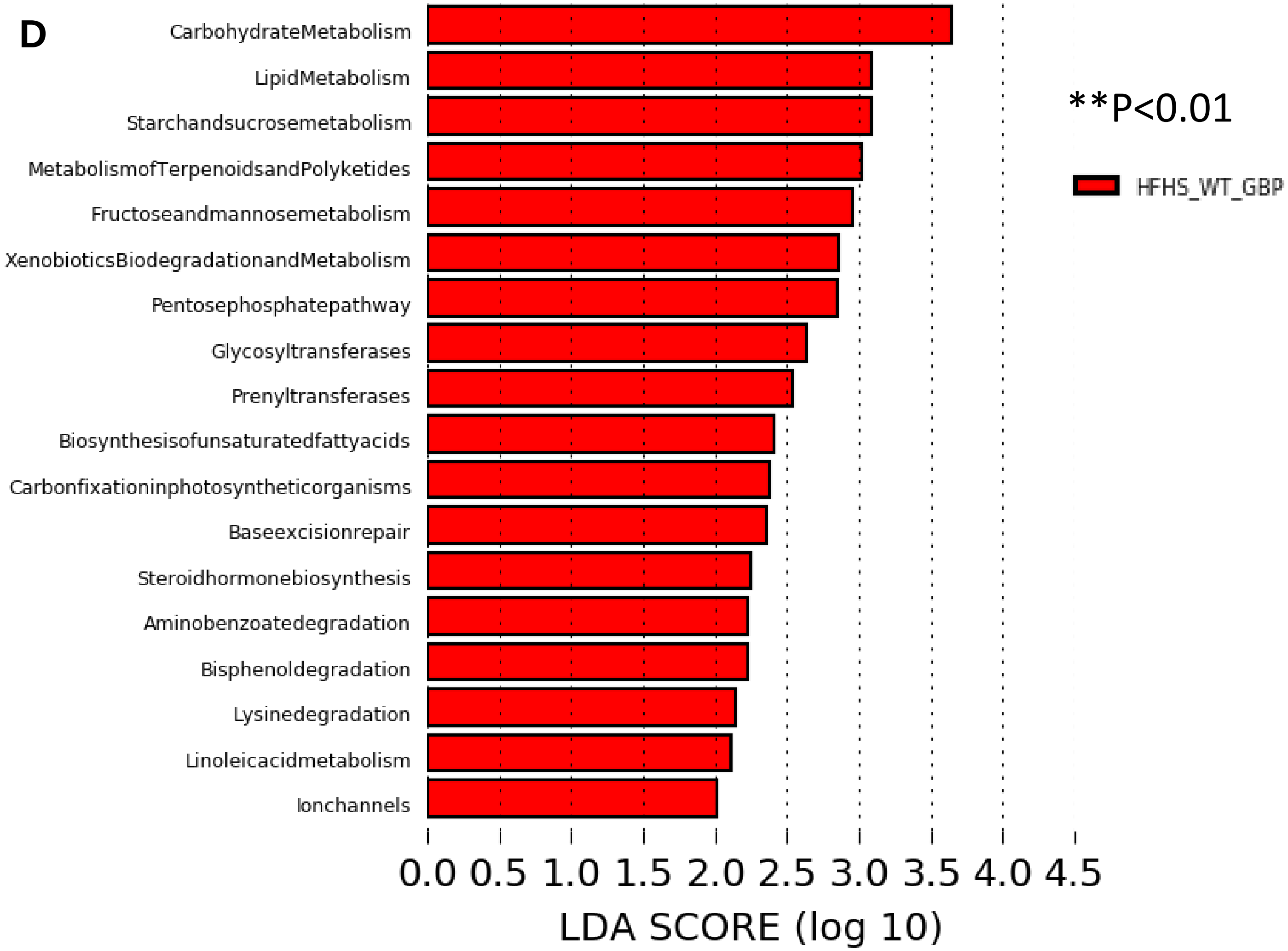
D
CarbohydrateMetabolism
LipidMetabolism
Starchandsucrosemetabolism
MetabolismofTerpenoidsandPolyketides
Fructoseandmannosemetabolism
XenobioticsBiodegradationandMetabolism
Pentosephosphatepathway
Glycosyltransferases
Prenyltransferases
Biosynthesisofunsaturatedfattyacids
Carbonfixationinphotosyntheticorganisms
Baseexcisionrepair
Steroidhormonebiosynthesis
Aminobenzoatedegradation
Bisphenoldegradation
Lysinedegradation
Linoleicacidmetabolism
Ionchannels
0.0 0.5 1.0 1.5 2.0 2.5 3.0 3.5 4.0 4.5
LDA SCORE (log 10)
**P<0.01
HFHS_WT_GBP

Suppl. Fig.11
